# ROS production and mitochondrial dysfunction driven by PU.1-regulated NOX4-p22^phox^ activation in Aβ-induced retinal pigment epithelial cell injury

**DOI:** 10.7150/thno.48064

**Published:** 2020-09-19

**Authors:** Junran Sun, Jieqiong Chen, Tong Li, Peirong Huang, Jie Li, Mengxi Shen, Min Gao, Yang Sun, Jian Liang, Xiaomeng Li, Yimin Wang, Yushu Xiao, Xiang Shi, Yifan Hu, Jingyang Feng, Huixun Jia, Te Liu, Xiaodong Sun

**Affiliations:** 1 Department of Ophthalmology, Shanghai General Hospital (Shanghai First People's Hospital), Shanghai Jiao Tong University School of Medicine, Shanghai, China.; 2 National Clinical Research Center for Ophthalmic Diseases, Shanghai, China.; 3 Shanghai Engineering Center for Visual Science and Photomedicine, Shanghai, China.; 4 Center for Advanced Vision Science, University of Virginia School of Medicine, Charlottesville, VA, USA.; 5 Department of Ophthalmology, Shanghai Municipal Hospital of Traditional Chinese Medicine, Shanghai University of Traditional Chinese Medicine, Shanghai, China.; 6 Department of Ophthalmology, Bascom Palmer Eye Institute, University of Miami Miller School of Medicine, Miami, Florida, USA.; 7 Department of Dermatology, Shanghai General Hospital, Shanghai Jiao Tong University School of Medicine, Shanghai, China.; 8 Shanghai Key Laboratory of Fundus Diseases, Shanghai, China.; 9 Shanghai Engineering Center for precise diagnosis and treatment of eye disease.; 10 Geriatric Institute of Chinese Medicine, Longhua Hospital, Shanghai University of Traditional Chinese Medicine, Shanghai, China.; 11 Department of Pathology, Yale University School of Medicine, New Haven, CT, USA.

**Keywords:** retinal pigment epithelial cells, amyloid β, age-related macular degeneration, PU.1, NADPH oxidases

## Abstract

**Rationale:** Amyloid β (Aβ) deposition, an essential pathological process in age-related macular degeneration (AMD), causes retinal pigment epithelium (RPE) degeneration driven mostly by oxidative stress. However, despite intense investigations, the extent to which overoxidation contributes to Aβ-mediated RPE damage and its potential mechanism has not been fully elucidated.

**Methods:** We performed tandem mass-tagged (TMT) mass spectrometry (MS) and bioinformatic analysis of the RPE-choroid complex in an Aβ_1-40_-induced mouse model of retinal degeneration to obtain a comprehensive proteomic profile. Key regulators in this model were confirmed by reactive oxygen species (ROS) detection, mitochondrial ROS assay, oxygen consumption rate (OCR) measurement, gene knockout experiment, chromatin immunoprecipitation (ChIP), and luciferase assay.

**Results:** A total of 4243 proteins were identified, 1069 of which were significantly affected by Aβ_1-40_ and found to be enriched in oxidation-related pathways by bioinformatic analysis. Moreover, NADPH oxidases were identified as hub proteins in Aβ_1-40_-mediated oxidative stress, as evidenced by mitochondrial dysfunction and reactive oxygen species overproduction. By motif and binding site analyses, we found that the transcription factor PU.1/Spi1 acted as a master regulator of the activation of NADPH oxidases, especially the NOX4-p22^phox^ complex. Also, PU.1 silencing impeded RPE oxidative stress and mitochondrial dysfunction and rescued the retinal structure and function.

**Conclusion:** Our study suggests that PU.1 is a novel therapeutic target for AMD, and the regulation of PU.1 expression represents a potentially novel approach against excessive oxidative stress in Aβ-driven RPE injury.

## Introduction

Age-related macular degeneration (AMD), the leading cause of blindness and low vision in elderly individuals, has become one of the most important global public health concerns. Thus far, there is no effective treatment for dry AMD, which accounts for approximately 80% of AMD cases. Drusen (amyloid β-containing deposits) formation is a characteristic pathological change of early AMD, the size of which is related to disease progression 1. Studies have shown that only 0.55% of early AMD patients are presented without drusen or with drusen < 125 μm in size developing progressive AMD within five years, while 11.11% of those with large drusen in both eyes have an accelerated incidence of progressive AMD within five years 2. Amyloid β (Aβ) is recognized as the primary pathological component in drusen, and its toxicity is associated with the induction of retinal pigment epithelial (RPE) cell senescence, apoptosis, oxidative stress, disruption of RPE cell junctions, as well as the loss of ability to sustain immune balance 3-6. Our previous studies have proposed that damaged RPE cells trigger a positive feedback loop to amplify fundus inflammation and accumulation of cytotoxic waste products, such as lipofuscin, leading to damage expansion to other retinal cells 3, 7. Thus, a detailed understanding of Aβ-driven RPE injury, especially in early AMD, may be vital to attenuate the cascade of fundus damages.

Following RPE impairment, Aβ triggers a proinflammatory and pro-angiogenic microenvironment in the fundus by activating the NLRP3 inflammasome and complement system and inhibiting platelet-derived growth factor (PDGF) 8-10. Both mechanisms are reported to play a central role in AMD progression, although the relative significance of these factors remains elusive. Also, the involvement of multiple targets, leading to complex changes in downstream signaling, hampers the identification of a generally accepted central mechanism and an effective therapeutic target for the Aβ_1-40_-mediated RPE damage. Based on proteomic analyses of the tandem mass tag (TMT) and liquid chromatography-tandem mass spectrometry (LC/LC-MS/MS); our results revealed enhancement of the oxidation-related pathway in Aβ-deposited RPE cells.

Recent studies have found that Aβ_1-40_ contributed to cellular dysfunction by activating reactive oxygen species (ROS) production 11. The accumulation of ROS under oxidative stress induces cellular senescence, lipid peroxidation, apoptosis, and endoplasmic reticulum (ER) stress and alters mitochondrial bioenergetics in RPE cells 3, 12, 13. The detailed mechanisms of oxidative stress in RPE cells and the extent to which it contributes to AMD have received little attention, although the intake of antioxidants, which can quench light-induced ROS, is the only recommended therapeutic strategy for dry AMD to date, as indicated in the Age-Related Eye Disease Study (AREDS) 14.

NADPH oxidase (NOX), an essential membrane-bound enzyme complex that triggers oxidative stress 15, is typically composed of the catalytic subunits gp91^phox^/NOX2 (encoded by CYBB), the transmembrane component p22^phox^ (encoded by CYBA), and the cytosolic components p47^phox^, p67^phox^, p40^phox^, and Rac1 or Rac2 (encoded by NCF1, NCF2, NCF4, RAC1, and RAC2, respectively). There are seven isozymes of NOX, depending on the identity of the catalytic subunit. A recent study demonstrated a link between Aβ-induced cellular damage and the activity of NOX isozymes in the oxidative stress response related to the TLR (Toll-like receptor)-mediated pathway 16. The NOX/ROS-mediated inflammatory response seems to be related to the activation of NF-κB, a crucial regulator of Aβ-induced retinal inflammation 7, 17, 18. Also, Aβ contributes to direct neuronal cell death by specifically activating NADPH oxidases 19. However, its distribution, activation, and specific regulatory mechanisms involved in the oxidative stress in RPE cells still need to be explored.

The transcription factor PU.1 (encoded by Spi1) of the E26 transformation-specific (ETS) family was shown to be the key regulator of NOX in Aβ-deposited RPE cells 20. PU.1 and ROS are closely associated, as PU.1 expression level was increased in H_2_O_2_-treated 3T3-L1 adipocytes 21. In the most recent large-scale genome-wide analysis, Huang et al. demonstrated a positive correlation between PU.1 expression and disease progression in Alzheimer's disease with Aβ deposition. The promoters of pathogenic genes are rich in the PU.1-binding motif, suggesting that PU.1 facilitates disease progression by transcriptionally modulating downstream genes 22.

In this study, we employed proteomic profiling to identify the mechanism of oxidative stress damage induced by PU.1 transcriptional regulation of NADPH oxidases in Aβ-deposited RPE cells. The results are highly significant for the protection of RPE cells from Aβ-mediated oxidative stress and maintenance of the fundus microenvironment stability and thus have promising potential for therapeutic development.

## Methods

### Patient criteria and analysis

This observational cross-sectional study involved 47 AMD patients, who have been followed up for disease progression at least for 5 years at the Shanghai General Hospital. All subjects presented with early-stage AMD in at least one eye from October 2009 to October 2019. All macular OCT scans taken during follow-up visits were reviewed retrospectively, and data regarding diagnosis, the affected eye, presence of drusen, AMD progression (the presence of advanced AMD at the last visit available was considered “progress”), and the location of drusen and advanced AMD lesions (choroidal neovascularization or geographic atrophy) were collected. The clinical definition of AMD was based on the AREDS 23. Patients were excluded by the following criteria: 1) diagnosis of advanced AMD at the first visit, 2) occurrence of other retinal diseases, and 3) no lesions at the foveal avascular zone (FAZ). Spectral domain OCT (SD-OCT) images were acquired with a Spectralis HRA + OCT platform (Heidelberg Engineering). All 3 × 3 mm OCTA images were obtained with an RTVue XR Avanti SD-OCT instrument (Optovue, Inc.) using a split-spectrum amplitude-decorrelation angiography (SSADA) algorithm.

This research was approved by the Ethics Committee and Institutional Review Board of Shanghai General Hospital. The clinical procedures conducted in this research followed the tenets of the Declaration of Helsinki.

### Amyloid oligomerization

Oligomeric Aβ_1-40_ peptide (Sigma) was prepared according to the manufacturer's instructions. Briefly, lyophilized Aβ_1-40_ peptides were dissolved in deionized distilled water (approximately 6 μg/μL). The solution was further diluted with phosphate-buffered saline (PBS) to a final concentration of 1.5 μg/μL (350 μM) before incubation for 4 days at 37 °C. The Aβ_1-40_ oligomers were stored at -20 °C before use.

### Animal model and treatment

Eight- to ten-week-old wild-type (WT) C57BL/6 mice (male) were provided by the Shanghai General Hospital, Laboratory Animal Center. The animals were raised in sterile enclosures. After anesthetization with 1.5% sodium pentobarbital (5 µL/g) intraperitoneally (i.p.), mice were administered a single unilateral IVL injection of oligomeric Aβ_1-40_ peptides (1.5 μg/2 μL) in PBS using a glass micropipette under a dissecting microscope (SM2000J, Olympus); PBS (2 μL) was injected into age-matched controls. Subretinal injection of 3 uL lentivirus vector (10^8^ TU/mL) was given 3 weeks before IVL injection. A lethal dose of pentobarbital was injected intraperitoneally for euthanasia. The Ethics Committee of Jiao Tong University, Shanghai, China, approved all animal experiments conducted in compliance with the Association for Research in Vision and Ophthalmology Statement for the Use of Animals in Ophthalmic and Vision Research.

### Primary mouse RPE cell isolation and culture

RPE cells were isolated from 3-week-old WT C57BL/6 mice for primary culture. The anterior portion of the eye and retina were removed gently with forceps, and RPE sheets were then peeled off and suspended under a dissecting microscope. Complete Dulbecco's modified Eagle's medium/Ham's F-12 medium (Gibco) was added to the collected single-cell suspension. The medium contained 10% fetal bovine serum, 1% nonessential amino acids, and 1% HEPES (Gibco). The cells were maintained at 37 °C in a humidified atmosphere of 5% CO_2_ and passaged every 3 days. Detailed information on primary RPE culture, confluency, maturity, epithelial phenotype, and polarization is provided in Supplementary Figure S1.

### TMT technology for quantitative proteomic analysis

RPE-choroid complex tissue samples obtained from C57BL/6 mice treated with PBS or Aβ_1-40_ (n=3 for each group) were used for proteomic analysis. Tissue sample preparation, trypsin digestion, TMT labelling using TMT10plex mass tag labelling kits and reagents (Thermo Fisher Scientific), and LC-MS/MS analysis were performed at the Shanghai Applied Protein Technology Co., Ltd. Parallel reaction monitoring (PRM) analysis was applied to determine the levels of protein expression gained through TMT analysis, as described in a previous study 24. Proteins significantly differentially expressed (absolute log_2_FC > 0.5 and P ≤ 0.05) in mice RPE-choroid complex are shown in Supplementary Table S1.

### Luciferase reporter assay

To determine the combination between promoters and PU.1, mouse NOX4 and CYBA gene promoters were ligated into luciferase reporter plasmid. Top-ranked binding sites in the promoter region predicted by FIMO from the MEME suite (http://meme-suite.org/doc/fimo.html) (NOX4: -1374 ~ -1355, CYBA: - 1960 ~ - 1941) were cloned into the KpnI-HindIII sites of the pGL6-TA-basic multi-cloning site 25. Luciferase assays were carried out in primary mouse RPE cells with the TransIT-X2 Dynamic Delivery System (Mirus Bio LLC) according to the manufacturer's instructions. Subsequently, 2.5 μg of pGL6-TA firefly luciferase reporter plasmids with the promoter of CYBA or NOX4 and mutation luciferase constructs were transfected into the cells. Firefly luciferase activity was detected using a luciferase assay kit (Beyotime).

### Measurement of mtROS

Cells cultured in 24-well plates were incubated with PBS or Aβ_1-40_ oligomer (2 μM) at 37 °C in an atmosphere of 5% CO_2_. After incubation for different lengths of time, the cells were treated with a working solution of MitoSOX Red (5 μM) (Invitrogen) for 20 min at 37 °C. Fluorescence was observed with a Leica TCS SP8 confocal laser scanning microscope (Leica TCS NT).

### Flow cytometry

Primary mouse RPE cells cultured in 6-well plates were incubated with PBS or Aβ_1-40_ oligomer. Cells were suspended in PBS containing 10 μM CM-H2DCFDA (5-(and-6)-chloromethyl-2',7'-dichlorodihydrofluorescein diacetate) (Invitrogen). After resuspension in the prewarmed buffer, RPE cells were incubated at 37 °C for 30 min, and fluorescence intensity, excited at 475 nm, was assessed by flow cytometry using CytoFLEX (Beckman Coulter) at an emission wavelength of 525 nm. Annexin V/PI staining was performed to detect apoptotic cell death using the Annexin V-FITC Apoptosis kit (Beyotime) according to the manufacturer's instructions. Flow cytometry (Beckman Coulter) was used to collect fluorescence intensity.

### Transmission electron microscopy (TEM) and hematoxylin & eosin (H&E) staining

Retinal RPE-choroid complexes were separated and fixed in 2.5% glutaraldehyde at 2 days and 4 days with or without Aβ_1-40_ treatment. The samples were then sent to Wuhan GuGe Biotechnology for TEM analysis. TEM images were used for mitochondrial ultrastructural observation. Retinal structures stained with H&E were evaluated by light microscopy. The inner nuclear layer, outer nuclear layer, and the total retinal thickness were measured at 4-6 points in each group within 200 μm from the optic disc (an area known to be of even thickness) on both sides at 400× magnification.

### Fundus photography

Fundus imaging of anesthetized C57BL/6 WT mice was carried out using the Phoenix MICRON IV system (Cold Spring Harbor Corp.) after pupil dilatation with 1% tropicamide (Alcon). Optic nerve heads marked the center of the images.

### Electroretinogram (ERG) acquisition

After dark adaptation for over 16 h, the mice were anesthetized with the intraperitoneal injection of 1.5% sodium pentobarbital. Mice with dilated pupils were placed on a stage, and then corneal contact electrodes, reference electrodes, and ground electrodes were placed on the mice. A Phoenix Ganzfeld System (Cold Spring Harbor Corp.) was used to record the scotopic a/b-wave at a stimulus intensity of 1.8-3.9 log cd sec/m^2^.

### Gene ontology (GO) and pathway enrichment analyses

DAVID (https://david‐d.ncifcrf.gov/), ClueGO 26, CluePedia 27, and Cytoscape 28 were used to analyze candidate differentially expressed protein (DEP) functions and pathway enrichment. GO term enrichment analysis was carried out using the DAVID database. Interrelation analysis between pathways was conducted using the ClueGO/CluePedia plugins, Cytoscape software, the Reactome pathway database, and the Reactome reactions database. A *P* value < 0.05 was used as the threshold value. GSEA software was used for GSEA of expression datasets (http://software.broadinstitute.org/gsea/index.jsp).

### Weighted gene co-expression network analysis (WGCNA)

The WGCNA package was used to construct a proteomic profile. Based on the adjacency matrix calculation, the topological overlap matrix (TOM) was used to identify protein co-expression modules, which then served as the input of hierarchical clustering analysis. A total of 15 modules were identified with a dynamic tree‐cutting algorithm and coded with different colors. A heatmap of the eigengene network was visualized.

### Immunofluorescence of RPE cells

Aβ_1-40_/PBS-treated mouse RPE-choroid complex and incubated primary RPE cells were fixed with 4% paraformaldehyde. The analysis was performed as previously described 7. Primary antibodies against the following were used for staining: 8-OHdG (1:100, sc-393871, Santa Cruz), cytochrome b245 light chain/p22^phox^ (1:500, ab75941, Abcam), and NOX4 (1:500, PA5-72816, Thermo Fisher Scientific).

### Protein-protein interaction (PPI) network establishment and modular analysis

We evaluated differentially expressed proteins (DEPs) and PPI information using the STRING database (available online: http://string-db.org). A functional association network of the identified proteins was generated by the STRING 8.3 webserver. Cytoscape software version 3.7.1 was applied to establish the PPI network.

### Oxygen consumption rate (OCR) assays

OCR measurements were performed using an XFe96 Extracellular Flux analyzer (Seahorse Bioscience) according to the manufacturer's instructions. Primary mouse RPE cells were seeded into an XFe96 polystyrene cell culture plate at 1 × 10^4^ cells per well and cultured overnight before the incubation with PBS or Aβ_1-40_ oligomer (2 μM) for different lengths of time. Prior to the assay, the growth medium was exchanged with the appropriate assay medium, and cells were incubated in a 37 °C/non-CO_2_ incubator for 1 h. Three baseline measurements and 3 response measurements were taken after the sequential addition of oligomycin, FCCP (Carbonyl cyanide 4-(trifluoromethoxy) phenylhydrazone), and rotenone/antimycin. OCRs were reported as absolute rates (pmol/min) and normalized against protein concentrations measured by the BCA method.

### Quantitative real-time PCR (qRT-PCR)

Total RNA was extracted from mouse RPE-choroid complexes or primary mouse RPE cells using the RNA Simple Total Kit following the manufacturer's protocol (Tiangen Biotech). RNA sample quality and concentration were detected using a NanoDrop 2000c spectrophotometer (Thermo Fisher Scientific). RT Master Mix (TaKaRa Bio, Inc.) was used to reverse-transcribe RNA to cDNA. Glyceraldehyde-3-phosphate dehydrogenase (GAPDH) was used to normalize the data. The primer sequences are shown in Supplementary Table S2. The RealPlex4 real-time PCR detection system (Eppendorf Co., Ltd.) was used to amplify cDNA with a program consisting of 40 cycles of amplification (Tm = 60 °C). The 2^-ΔΔCt^ method was used to calculate the gene expression level of each mRNA.

### Western blotting and co-immunoprecipitation (Co-IP)

Total protein was extracted from RPE cells and RPE-choroid tissues and lysed. Aliquots of each sample were resolved on 10% SDS-PAGE gels and then transferred to polyvinylidene difluoride membranes (Merck Millipore). After blocking, washing in the buffer (5% non-fat milk powder dissolved in Tris-buffered saline Tween-20 (TBST)) and washing again, the membranes were incubated with primary antibodies at 4 °C overnight. The membranes were washed three times with TBST. Horseradish peroxidase-conjugated secondary antibodies (1:3000, Proteintech) were used to probe the membranes for 1 h. Then, the washed membranes were finally exposed to a molecular imaging system (Amersham Imager 600, GE Healthcare). Primary antibodies against the following were used: cytochrome b245 light chain/p22^phox^ (1:2000, ab75941, Abcam), NOX4 (1:2000, PA5-72816, Thermo Fisher Scientific), and PU.1 (1:1000, #2258, CST).

A Pierce Co-Immunoprecipitation Kit (Rockford) was used to carry out Co-IP according to the manufacturer's instructions. The antibodies used for immunoprecipitation were the same as used for Western blot analysis as described above.

### ChIP assay

The ChIP assay was performed, as previously described 7. Briefly, primary mouse RPE cell lysate was sonicated to shear DNA into 200-500 bp fragments, and a ChIP-grade antibody against PU.1 (#2258, CST) was used for immunoprecipitation. The ChIP product was quantified using RT-PCR with specific primers for the regulated gene promoters. ChIP primer sequences are listed in Supplementary Table S2.

### Knockdown assays

To perform lentivirus-mediated shRNA interference, pLKD-CMV-R&PR-U6-shRNA vectors (Heyuan) carrying shRNA against Spi1 or scramble shRNA were constructed. The sequence of the oligonucleotide encoding shRNA targeting Spi1 used in mouse RPE cells was 5'- GGATGTGCTTCCCTTATCAAA -3'. The siRNAs were designed and synthesized by Hippobio.

### Statistical analysis

The number of samples and details of the statistical tests conducted are given in the corresponding figure legends. GraphPad Prism 6 (GraphPad Software, Inc.) software was used for all statistical analyses except for Fisher's exact test, which was conducted with MATLAB (The MathWorks, Inc.). Differences with *P* < 0.05 were considered to be statistically significant.

## Results

### Drusen are positively related to AMD progression

Since drusen are related to AMD progression, we examined 47 eyes from patients with early or intermediate AMD who received macular optical coherence tomography (OCT) scans at their first visit to Shanghai General Hospital from 2009 to 2019. Among these 47 patients, 23 showed no progress at the last follow-up examination, while 24 patients presented with advanced AMD. Notably, 23 of these 24 early AMD patients with drusen (95.8%) developed advanced AMD within 5 years, and only 1 patient without drusen (4.2%) progressed to advanced stage AMD (P < 0.001, Fisher's exact test) (Table 1). The locations of choroidal neovascularization (CNV) and drusen among these 23 patients observed by OCT were carefully reviewed. CNV or geographic atrophy (GA) lesions, indicating the advanced stage of AMD, were identified consistent with drusen deposition with RPE alteration and degeneration in 20 patients (87.0%) (Supplementary Figure S2A). To further investigate the role of drusen in AMD pathogenesis, 3 of 47 patients with early-stage dry AMD received optical coherence tomography angiography (OCTA), which showed an association of drusen with choriocapillaris flow impairment (Supplementary Figure S2B-C). These findings indicated that drusen is likely related to RPE damage and degeneration, exacerbating AMD progression.

### The Aβ_1-40_-deposited fundus reveals AMD-like pathologies

**Figure 1:**
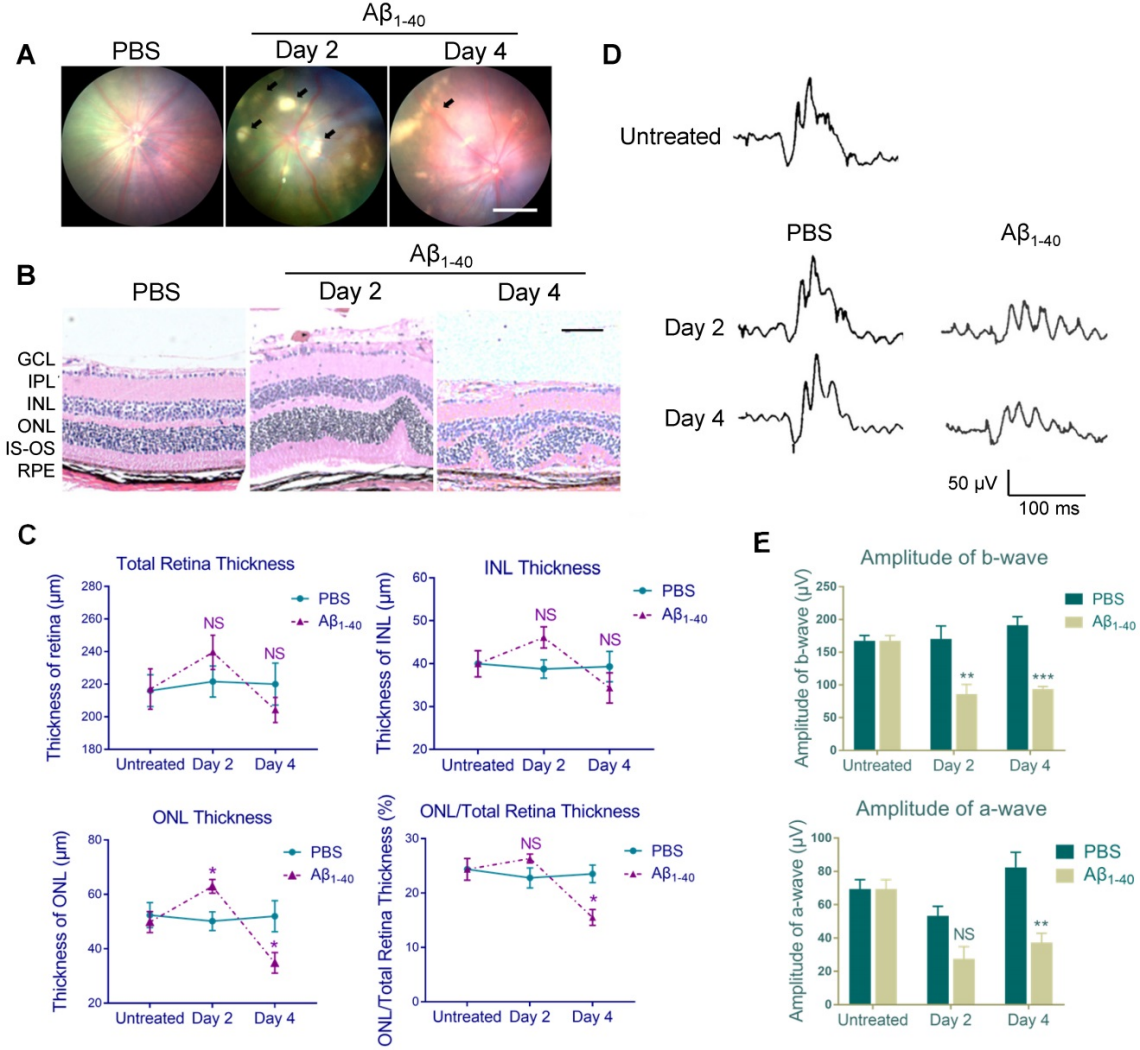
** Aβ_1-40_-injected mice exhibit AMD-like pathologies.** (**A**) Fundus photography of the eyes of PBS- and Aβ_1-40_-injected WT mice. Multiple patchy deposits (black arrows) were found in the Aβ_1-40_-treated group at 2 and 4 days following the injection. Scale bar = 500 µm. (**B**) Representative images of retinal sections of the Aβ_1-40_-treated group within 200 µm from the optic disc on days 0, 2, and 4. Scale bar = 100 µm. (**C**) Changes in the thickness of the INL, ONL, and total retina and the ratio of ONL/total retina thickness. The ONL thickness was significantly increased after 2 days of Aβ_1-40_ deposition and significantly decreased after 4 days compared with the PBS-treated controls. GCL: ganglion cell layer; IPL: inner plexiform layer; INL: inner nuclear layer; ONL: outer nuclear layer; IS: inner segment; OS: outer segment; RPE: retinal pigment epithelium. (**D**) Average traces from ERG recordings and (E) maximum scotopic ERG a- and b-wave amplitudes from mice at 0, 2, and 4 days post-injection. All results are presented as the mean ± SEM; n = 4-5 for each group. NS = nonsignificant, *P < 0.05. **P < 0.01, ***P < 0.001, Student's *t*-test, SEM: standard error of the mean.

Based on our previous study, showing that the intravitreal (IVL) oligomeric Aβ_1-40_ injection in mice resulted in histopathologic and functional manifestations similar to those in AMD 7, we conducted fundus imaging to identify retinal changes 2 and 4 days after the injection. No apparent pathology was noted in PBS-treated WT C57BL/6 mice. However, multiple patchy deposits were observed in the eyes of Aβ_1-40_-injected mice due to the subretinal accumulation of Aβ_1-40_ oligomers. The deposits were visible under the retinal vessels as early as 2 days following Aβ_1-40_ injection and lasted for at least 4 days (Figure 1A).

Histological analysis of PBS-treated controls revealed a normal retinal morphology, while sections from the Aβ_1-40_-treated group showed waved or whorled retinas with RPE pigmentary changes and disruption of the inner and outer segments (Figure 1B). There was no significant difference in the thickness of the total retina and inner nuclear layer (INL) between the two groups, which was consistent with a previous study 9. Furthermore, the outer nuclear layer (ONL) was thicker on day 2 and became thinner on day 4 in Aβ_1-40_-treated mice than PBS-treated controls. The ONL thickness/total retinal thickness ratio indicated acute injury of the Aβ_1-40_-treated retina, followed by the progressive loss of photoreceptor cells (Figure 1C). Subsequently, we quantified retinal responses to photic stimulation using electroretinogram (ERG). The b-wave amplitudes (photoreceptor-stimulated bipolar cell responses) of mice injected with Aβ_1-40_ on day 2 post-injection were significantly decreased compared to the PBS-injected controls, and the downregulation became more pronounced on day 4. A substantial reduction in a-wave amplitudes (primarily photoreceptor-derived) was noted on day 4, revealing retinal function damage (Figure 1D-E). Thus, retinal structural changes highly correlated with the ERG findings. These results suggested that mice intravitreally injected with Aβ_1-40_ can serve as a model of RPE and photoreceptor degeneration, and the 4-day post-injection time point is most suitable for following retinal responses.

### Proteome analysis with TMT-based isobaric labeling indicates an oxidant-related pathway in Aβ_1-40_-deposited RPE cells

**Figure 2:**
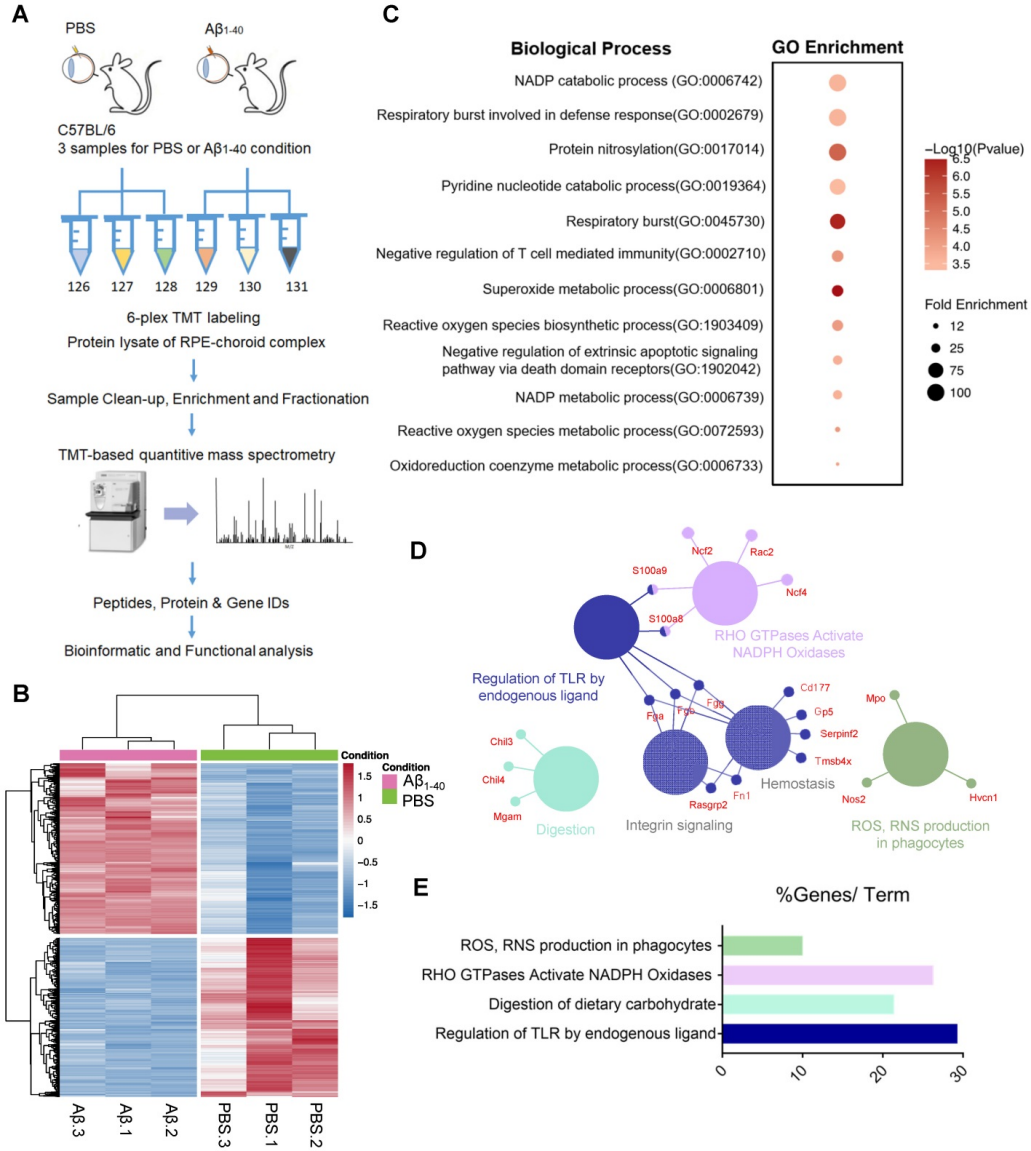
** Proteomic analysis of the RPE-choroid complex from Aβ_1-40_- or PBS-treated mice using TMT-based isobaric labeling.** (**A**) Schematic of the workflow of TMT and multi-omics analyses. (**B**) Heatmaps of differentially expressed proteins (DEPs) (-0.5 < log_2_FC < 0.5 and *P* ≤ 0.05) excluding cytoskeleton-related proteins (red, upregulation; blue, downregulation; n=3). (**C**) GO enrichment analysis of the top 100 most highly upregulated DEPs; major GO terms were enriched in oxidation-related biological processes. (**D, E**) Functional pathway analysis with the ClueGO and CluePedia Cytoscape plugins with the Reactome pathways database showing overrepresented pathways related to ROS production.

To determine how Aβ_1-40_ deposition affects RPE cells and delineate the proteomic landscape, we analyzed protein expression profiles of the RPE-choroid complex from mice intravitreally injected with Aβ_1-40_ using TMT-based quantitative mass spectrometry (MS) 29 with PBS-treated mice serving as a control (Figure 2A). The experiment was performed in three replicates for each group (Figure 2B). We identified 10660 and 4243 nonredundant peptides and proteins, respectively. It has previously been shown that Aβ can trigger a reorganization of the cytoskeletal network 30. In this study, we examined the regulatory mechanisms downstream of Aβ_1-40_ and found cytoskeleton-related proteins to be the most abundant by MS analysis. Supplementary Figures S3A-B displays the distribution of cytoskeletal proteins. To avoid a potential bias towards the cytoskeletal protein family, we first removed cytoskeleton-related proteins (according to GO: 0044430-cytoskeleton) from the input list (113 genes in total), identifying 1069 DEPs between the Aβ_1-40_-treated and PBS-treated groups (Figure 2B). A total of 554 upregulated genes and 515 downregulated genes in the Aβ_1-40_-treated group compared with the control group (log_2_ (fold change [FC]) > 0.5 or < -0.5 and P ≤ 0.05) were identified (Supplementary Table S1).

**Figure 3:**
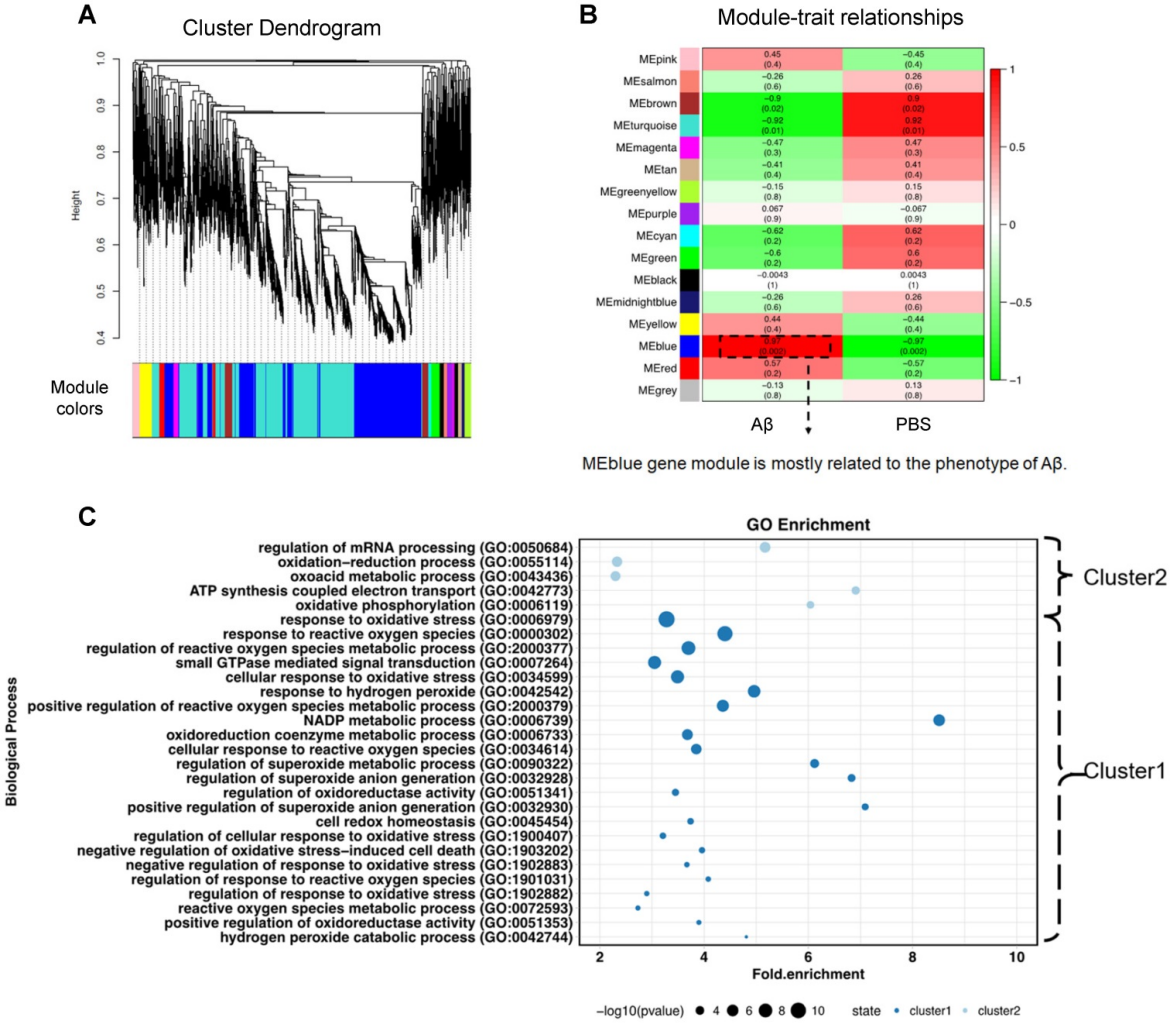
** Weighted gene co-expression network analysis (WGCNA) to identify Aβ_1-40_-related functional modules.** (**A**) Hierarchical clustering dendrogram demonstrating color-coded gene co-expression modules (ME). The MEblue gene module had the strongest correlation with the phenotype following Aβ_1-40_ treatment (*r* = 0.97; *P* = 0.002). (**B**) Relationships between modules and Aβ_1-40_ traits and the corresponding P values. Correlations are marked by colors as indicated in the color legend on the left. (**C**) GO enrichment analysis of genes in the MEblue module was divided based on expression level into “cluster 1” and “cluster 2” showing the enrichment of oxidation-related pathways.

The top 100 most strongly upregulated DEPs were selected according to log_2_FC, and gene ontology (GO) enrichment analysis was performed using an online tool (PANTHER: www.pantherdb.org). As displayed in Figure 3C, oxidation-related GO ontology was enriched. Network analysis of these proteins using the ClueGO and CluePedia plugins of Cytoscape with the Reactome pathways database showed that NADPH oxidases and ROS production were affected by Aβ_1-40_ treatment (Figure 2D-E) 31.

To obtain a universal description of co-expression patterns within proteins highly related to Aβ_1-40_, weighted gene co-expression network analysis (WGCNA) 32 was applied. A total of 15 protein modules covering the entire input dataset were identified. The hierarchical clustering dendrogram showed co-expressed proteins that were highly correlated (Figure 3A). Identification of the Aβ_1-40_-related gene module by WGCNA, the blue eigengene gene module (MEblue), containing 1393 proteins, was mostly related to the phenotype following Aβ_1-40_ treatment (with the highest correlation coefficient = 0.97 and p = 0.002, Figure 3B). The heatmap (Supplementary Figure S3C) displays the expression pattern of genes in the MEblue, which were divided into “cluster 1” (985 proteins) and “cluster 2” (411 proteins) based on their expression. Further, GO enrichment analysis of clusters 1 & 2 indicated that proteins in the MEblue module were enriched in oxidant-related pathways (Figure 3C) and play an important role in regulating Aβ_1-40_-induced RPE damage through the production of ROS, causing oxidative stress in RPE cells.

### Aβ_1-40_ treatment impairs the mitochondrial function of RPE cells and induces ROS production

**Figure 4:**
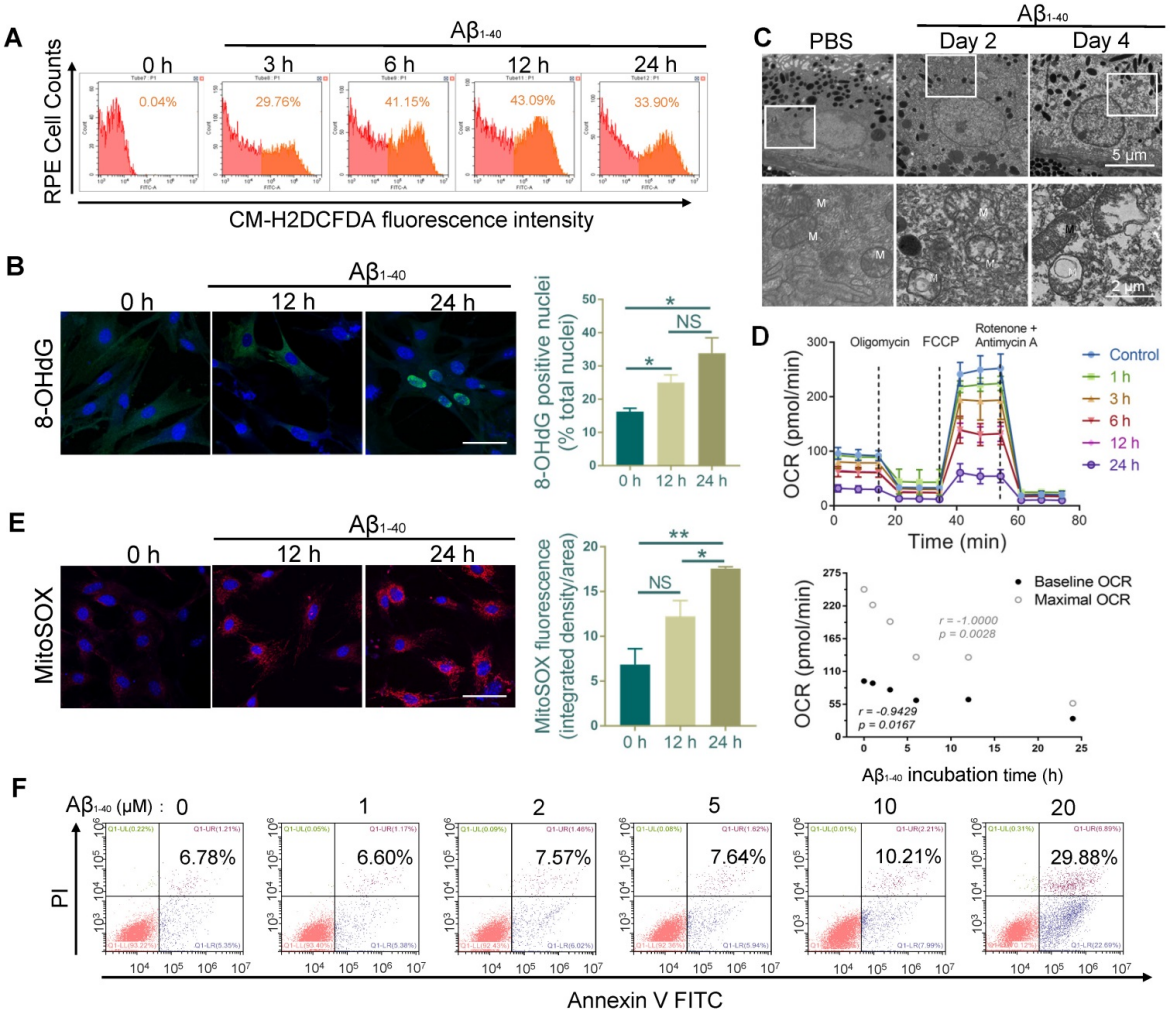
** RPE cell oxidation and impaired mitochondrial functions after Aβ_1-40_ treatment.** (**A**) Primary mouse RPE cells were treated with Aβ_1-40_ (2 µM) for 0, 3, 6, 12 h, and 24 h before incubation with CM-H2DCFDA. Flow cytometry was used to detect the fluorescence intensity. (µ) Representative frozen RPE cell sections stained at 0 h, 12 h, and 24 h after Aβ_1-40_ treatment for immunoreactive 8-hydroxy-deoxyguanosine (8-OHdG, green). Nuclear 8-OHdG expression was quantitated as a percentage of the total nuclei. Scale bar = 50 µm. (**C**) TEM images of changes in the morphology and mitochondria of RPE cells at 2 and 4 days after intravitreal Aβ_1-40_ injection. The lower parts of the images are magnified portions of the corresponding upper images (enclosed in white boxes). M, mitochondria; scale bar = 5 µm (upper portion) and 2 µm (lower portion). (**D**) The OCRs of primary mouse RPE cells after incubation with Aβ_1-40_ for different time periods (0 h, 1 h, 3 h, 6 h, 12 h, and 24 h) in the presence of oligomycin, FCCP, and rotenone were measured using a Seahorse XF24 analyzer (n=5). Average basal and maximal OCRs were calculated as the mean of the measurements at 3 time points. The basal and maximal OCRs were negatively related to the Aβ_1-40_ incubation time. (**E**) Representative confocal microscopy images of primary mouse RPE cells stained with MitoSOX Red at 0 h, 12 h, and 24 h after Aβ_1-40_ treatment. Scale bar = 50 µm. (F) Apoptosis analysis in primary mouse RPE cells after incubation with Aβ_1-40_ at different concentrations (0 µM, 1 µM, 3 µM, 6 µM, 12 µM, and 24 µM) was performed by flow cytometry with annexin V/PI double staining. All data are presented as the mean ± SEM; NS = nonsignificant, *P < 0.05, **P < 0.01. Student's t-test (B, E) and Spearman's correlation analysis (D) were performed using GraphPad Prism software.

The multi-omics analysis showed significant involvement of the oxidant-related pathway in Aβ_1-40_-induced damage, suggesting the accumulation of excess ROS. We monitored the total ROS levels in primary mouse RPE cells by CM-H2DCFDA fluorescence using flow cytometry. The increase in CM-H2DCFDA fluorescence intensity was observed at 3 h following Aβ_1-40_ treatment and peaked at 12 h (Figure 4A). To further confirm the exacerbation of oxidative stress from increased ROS, Aβ_1-40_-treated RPE cells were stained at different time points with an oxidative stress marker, 8-hydroxy-2'-deoxyguanosine (8-OHdG). The percentage of 8-OHdG-positive nuclei was significantly higher in the Aβ_1-40_-treated cells after 12 h and 24 h than in the untreated cells (Figure 4B). Previous studies have shown that the mitochondria and the NADPH oxidase system are the two major sources of ROS overproduction induced by Aβ_1-40_ 33-35.

We analyzed PBS- and Aβ_1-40_-treated RPE-choroid complexes by TEM. A disorganized mitochondrial structure was observed in the RPE cells (Figure 4C), suggesting that Aβ_1-40_ deposition impaired RPE mitochondrial function. To confirm this observation, the real-time OCR, a measure of mitochondrial function, was measured in Aβ_1-40-_treated primary mouse RPE cells at different time points. The baseline and maximal OCRs were suppressed by treatment with Aβ_1-40_ in a time-dependent manner (Figure 4D). At 24 h following Aβ_1-40_ treatment, the OCR during maximal oxygen consumption was decreased by ∼80%, indicating a reduced respiratory capacity. When mitochondrial ROS (mtROS) levels were detected using MitoSOX Red, a mitochondrial superoxide indicator, a significant increase was detected at 24 h after Aβ_1-40_ treatment compared to that at 0 h (Figure 4E).

To determine if ROS elevation and mitochondrial damage are features of Aβ-mediated RPE apoptosis, Annexin V-FITC/PI double staining was performed in primary mouse RPE cells at 12 h following Aβ_1-40_ treatment (Figure 4F). Compared to the untreated group, the apoptosis rate did not exhibit any significant difference following 2 μM Aβ_1-40_ treatment. However, mitochondrial damage, dysfunction, and mtROS levels were increased peaking at 24 h post-treatment. In contrast, the total ROS level peaked at 12 h following Aβ_1-40_ treatment and then declined at 24 h. This gap in time of the total ROS and mtROS levels reaching the respective peak levels supported the presence of different ROS producers in Aβ_1-40_-treated RPE cells, suggesting that damaged mitochondria are the main source of ROS in the late stage of Aβ-mediated RPE injury.

### NOX4 and its family members are the key proteins involved in Aβ_1-40_ deposition-induced oxidative stress

**Figure 5:**
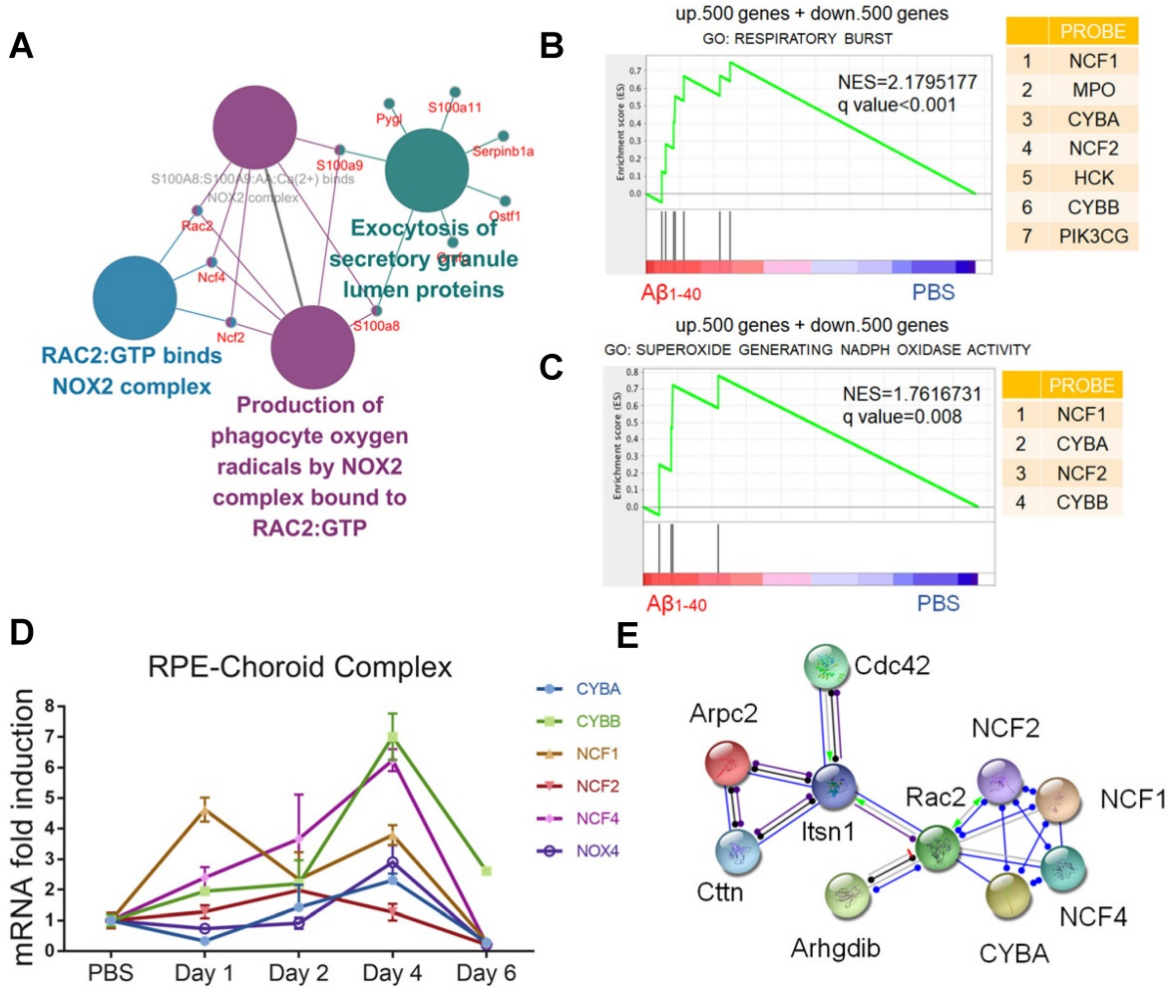
** NADPH oxidases are related to oxidative stress in Aβ-deposited cells.** (**A**) ClueGO/CluePedia network analysis to visualize pathways and their related genes. The hub genes (proteins) are marked in different colors. (**B, C**) GSEA of gene sets related to respiratory burst (GO: 0045730) and superoxide-generating NADPH oxidase activity (GO: 0016175) were performed. The 500 most highly up- and downregulated genes were merged and used as the input gene list. NADPH oxidase components (NCF1, CYBA, NCF4, and CYBB) were significantly upregulated in both pathways. NES: normalized enrichment score. (**D**) The mRNA levels of CYBA, CYBB, NOX4, NCF2, and NCF4 at different time points after exposure to Aβ_1-40_ were evaluated by qRT-PCR. The mRNA levels were standardized to those of a housekeeping gene. (**E**) High confidence interaction network within proteins of the NADPH oxidase complex generated by the STRING database (interaction score: 0.9).

To further investigate the source of Aβ_1-40-_induced ROS and identify hub proteins involved in oxidation-related pathological processes and the enrichment of the top 100 upregulated proteins, we applied ClueGO analysis with the Reactome pathways database. Figure 5A displays only terms with *P* < 0.05. We found that the NOX regulatory subunits p47^phox^ (NCF1), p40^phox^ (NCF4) and Rac2 (RAC2) were hub proteins that mapped to both “RAC2: GTP binds NOX2 complex” and “Production of phagocyte oxygen radicals by NOX2 complex bound to RAC2: GTP” pathways.

Gene set enrichment analysis (GSEA: http://www.broadinstitute.org/gsea/) of the 500 up- and downregulated genes was then carried out. The results confirmed that NOX, including the catalytic and cytosolic subunits, was involved in Aβ_1-40_-induced oxidative stress (Figure 5B-C). Consistent with the *in vivo* studies, CYBA, CYBB, NOX4, NCF2, and NCF4 mRNA levels were upregulated in RPE cells 4 days after exposure to Aβ_1-40_ and 1 day after exposure to NCF1 (Figure 5D). NOX4 silencing effectively downregulated both ROS and mtROS levels (Supplementary Figure S5A-D). To further investigate the regulators of NADPH oxidases and identify possible intervention targets, the STRING database (http://string-db.org/) was employed to detect functional partnerships and upstream networks of NADPH oxidases. We found the NOX complex interactions to be exclusively within its family members (Figure 5E).

### PU.1 expression is essential for NADPH oxidase transcription

**Figure 6:**
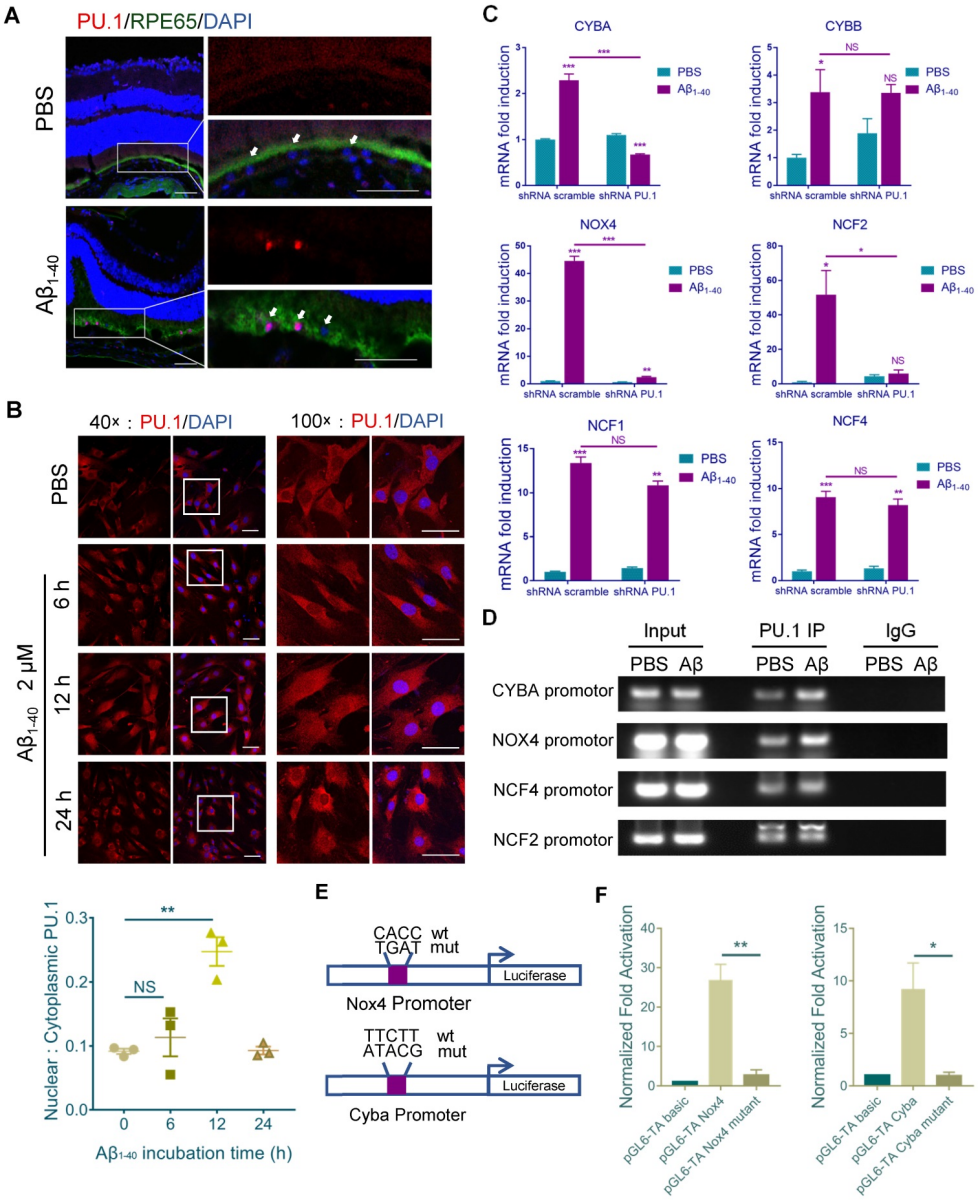
** PU.1 is a key transcriptional regulator of NADPH oxidase.** (**A**) Representative confocal microscopy images of frozen retinal sections for RPE cells (arrows) stained with PU.1 (red) at 4 days post-injection. Scale bar = 50 µm. (**B**) Immunoreactivity for PU.1 in the nuclei and quantification of nuclei/cytoplasm ratios of PU.1 staining in Aβ_1-40_-treated primary mouse RPE cells at 0, 6, 12, and 24 h. Images labeled “100×” are magnified portions of the images labeled “40×” (white boxes). Scale bar = 50 µm. (**C**) Primary mouse RPE cells were transfected with lentivirus carrying scramble shRNA and PU.1 shRNA for 24 h. NADPH oxidase subunit mRNA levels were evaluated by qRT-PCR and standardized to those of a housekeeping gene. (**D**) ChIP analysis of the association of PU.1 with the CYBA, NOX4, NCF4, NCF2, and CYBB gene promoters was conducted 12 h after Aβ_1-40_ stimulation. The immunoprecipitated DNA was then analyzed by semiquantitative PCR using promoter-specific PCR primers. Input DNA was used as a normalization control. (**E**) Schematic diagram representing the binding sites of different gene promoters and corresponding mutants. (**F**) pGL4-TA vectors containing binding sites for the Cyba and Nox4 promoters were transfected into primary RPE cells. Luciferase activity was measured 12 h after transfection and compared to that in cells transfected with pGL6-TA basic. All data are presented as the mean ± SEM; n = 3 for each group. NS = nonsignificant, *P < 0.05, **P < 0.01, ***P < 0.001, Student's *t*-test.

Sequence analysis (http://alggen.lsi.upc.es) was used to predict common transcriptional factors within the 5' upstream promoter regions of NCF1, NCF2, NCF4, NOX4, CYBB, and CYBA (~2 kb). Among the candidate transcriptional factors, PU.1/Spi1 and C/EBPβ were selected for further assessment since both were increased at both mRNA and protein levels after Aβ_1-40_ treatment of RPE cells (Supplementary Figure S4A-B). Furthermore, the JASPAR database was used to identify the transcription binding site motifs in PU.1 and C/EBPβ (http://jaspar.genereg.net/). Our analysis revealed a statistically significant higher PU.1 motif affinity for the NADPH oxidase promoter than the C/EBPβ motif, which was confirmed by ChIP analysis (Supplementary Figure S4C). Also, a strong fluorescence signal for PU.1 was observed in the nuclei of RPE cells (marked by RPE65) at 4 days after Aβ treatment (Figure 6A). PU.1 nuclear translocation was also noted upon incubation of primary mouse RPE cells with Aβ_1-40_ for 6 h, and the signal was greater than that in PBS-treated cells at 12 h, accompanied by an increase in nuclear/cytoplasmic ratio of PU.1 staining (Figure 6B).

When primary mouse RPE cells were treated with Aβ_1-40_ for 12 h. mRNA levels of CYBA, NOX4, and NCF2 in the PU.1-knockdown group were decreased, especially for CYBA and NOX4, compared with the group treated with scramble small hairpin RNA (shRNA) (Figure 6C). The interference effect of lentivirus shRNA is shown in Supplementary Figure S4D. The DNA fragments were immunoprecipitated using anti-PU.1 antibody and subjected to semiquantitative PCR. ChIP analysis showed that Aβ_1-40_ deposition was accompanied by an increase in PU.1 binding to the promoter regions of CYBA and NOX4 *in vitro* (Figure 6D). Luciferase reporter vectors containing the top binding sites predicted by FIMO from the MEME suite (http://meme-suite.org/doc/fimo.html) for NOX4 (-1374 ~ - 1355) and CYBA (-1960 ~ -1941) were generated to verify transcriptional activity. The luciferase activity in cells transfected with the pGL6-TA Nox4 vector was approximately 27-fold higher than in cells transfected with the pGL6-TA basic vector. The luciferase activity in cells transfected with the pGL6-TA Cyba vector was approximately 9-fold higher than in cells transfected with the pGL6-TA basic vector (Figure 6E-F). These results confirmed that PU.1 could transcriptionally regulate NOX4 and CYBA directly.

**Figure 7:**
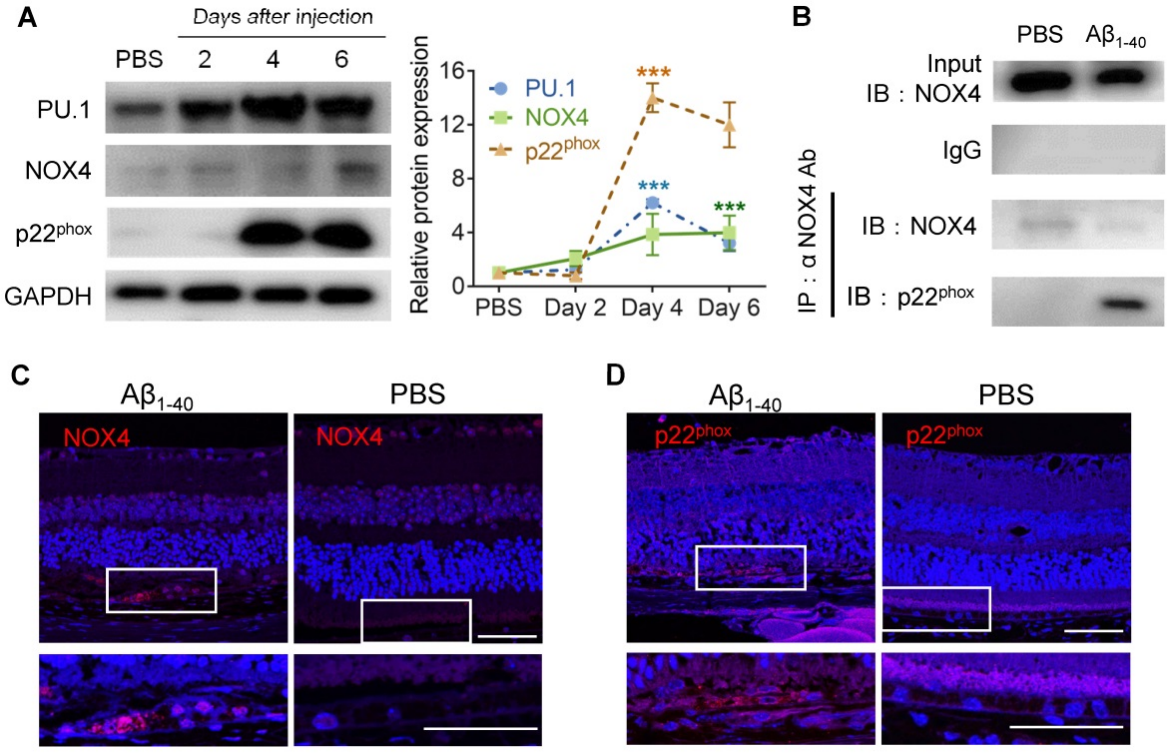
** PU.1 upregulation is concomitant with NOX4- p22^phox^ complex synthesis.** (**A**) PU.1, p22^phox^ (CYBA), and NOX4 expression and quantification in mouse RPE cells after 2, 4, and 6 days of Aβ_1-40_ treatment. (**B**) Co-IP of NOX4 and p22^phox^ (CYBA) at 4 days after the injection of Aβ_1-40_ or PBS. (**C, D**) Retinal sections were immuno-stained for NOX4 (C) and p22^phox^ (D) at 4 days post-injection. The lower parts of the images are magnified portions of the RPE layer from the upper parts of the images (white boxes). Scale bar = 50 µm; all data are presented as the mean ± SEM; n = 3 for each group. NS = nonsignificant, ***P < 0.001, Student's *t*-test.

Furthermore, the expression level of NOX4 was elevated on day 6, while p22^phox^ (CYBA) was elevated on day 4 and day 6 in the RPE-choroid complex in which PU.1 was upregulated 2 days after Aβ_1-40_ injection (Figure 7A). Co-IP analysis proved that NOX4 interacted with p22^phox^ (Figure 7B), and its activation was reported to increase intracellular ROS 36. Retinal section staining further confirmed NOX4 and p22^phox^ expression in Aβ_1-40_-treated RPE cells (Figure 7C-D). Thus, PU.1 is a key transcriptional regulator of NOX, and NOX4/p22^phox^ may play a role in Aβ_1-40_-induced ROS production, which was not reported in previous studies.

### PU.1 facilitates Aβ_1-40_-induced RPE degeneration

**Figure 8:**
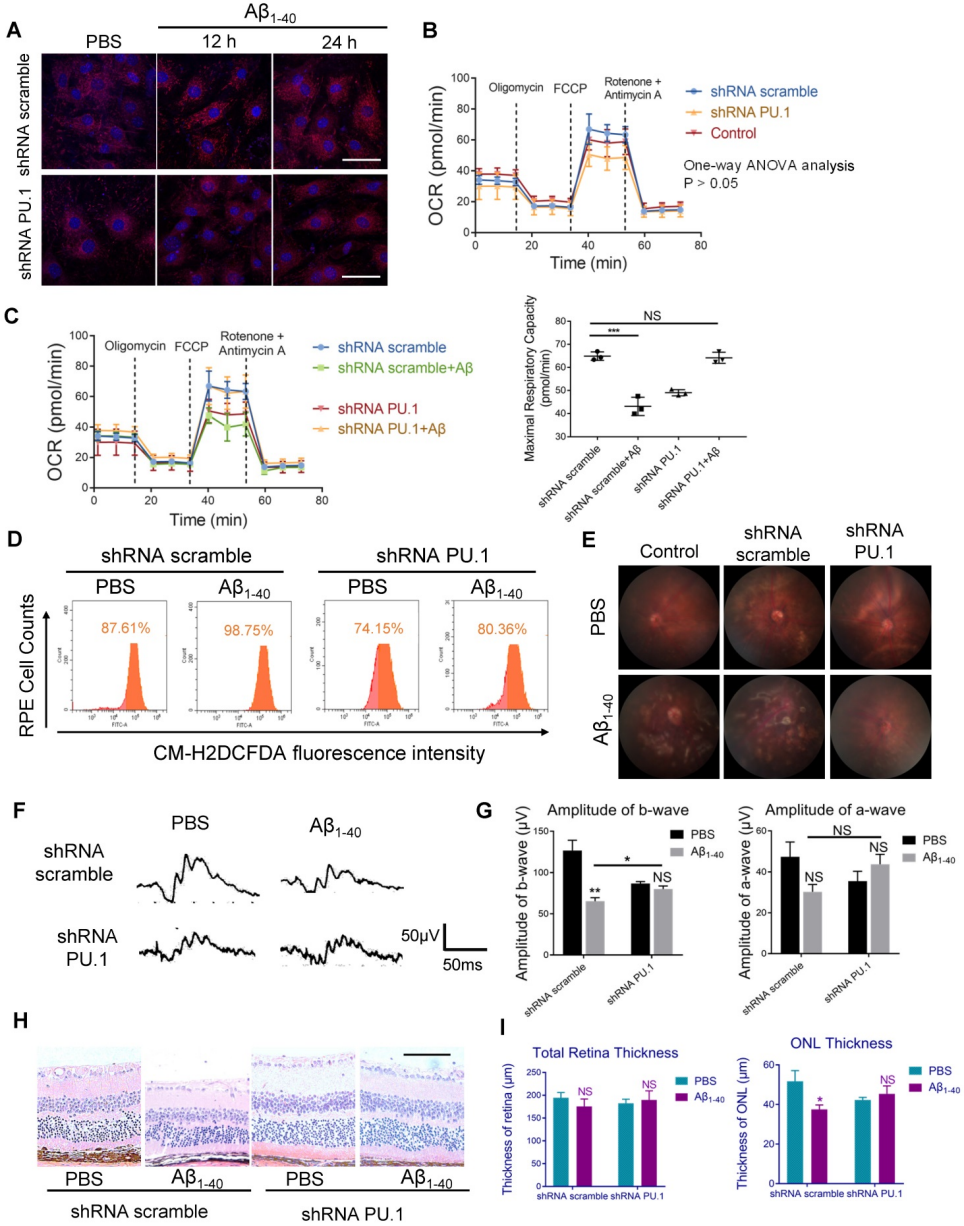
** PU.1 regulates RPE oxidative stress, causing Aβ_1-40_-induced RPE degeneration.** (**A**) Representative images of MitoSOX Red-stained primary mouse RPE cells transduced with PU.1/scramble shRNA lentiviruses at 0 h, 12 h, and 24 h after Aβ_1-40_ treatment. Scale bar = 50 µm. (**B**) The OCRs of primary mouse RPE cells in the scramble shRNA-treated group, PBS-treated (control) group, and PU.1 shRNA-treated group had no difference. *P* > 0.05 for all time points from 1 to 75 min (one-way ANOVA). (**C**) OCRs of the PU.1 shRNA-treated group and scramble shRNA-treated group at 24 h after Aβ_1-40_ treatment. The average maximal OCR/antimycin- and rotenone-induced OCR was calculated as the mean of the values at 3 time points (40-54 min); n = 3. (**D**) The CM-H2DCFDA fluorescence intensity was detected in primary mouse RPE cells transduced with lentivirus carrying PU.1 shRNA for 72 h followed by Aβ_1-40_ incubation for 12 h. (**E**) Fundus photography of the eyes of mice subretinally injected with lentiviruses carrying scramble shRNA or PU.1 shRNA 4 days after Aβ_1-40_ or PBS treatment. (**F, G**) Representative traces from ERG recordings of mice subretinally injected with lentivirus 4 days after the injection of Aβ_1-40_. The corresponding a- and b-wave amplitudes are shown in (G). (**H**) Representative retinal sections within 200 µm from the optic disc in mice subretinally injected with lentiviruses carrying scramble shRNA and PU.1 shRNA 4 days after Aβ_1-40_ or PBS treatment. Scale bar = 50 µm. (**I**) Changes in the thickness of the ONL and the total retinal thickness in (H). All data are presented as the mean ± SEM; NS = nonsignificant, *P < 0.05, ***P < 0.001, Student's *t*-test (C, G and I) and one-way ANOVA (B) were performed using GraphPad Prism software.

To examine whether PU.1 affected Aβ_1-40_-induced oxidative stress, lentivirus-mediated shRNA targeting PU.1 and control scrambled shRNA were constructed. shRNA PU.1 lentiviral transfection markedly decreased Aβ_1-40_-stimulated total ROS and mtROS levels in primary mouse RPE cells (Figure 8A, D). Also, the OCR, which was impaired by Aβ_1-40_, did not show a significant change in shRNA PU.1 lentivirus-transduced RPE cells (Aβ_1-40_ + PU.1 shRNA group) compared with scramble shRNA-transduced RPE cells at 24 h following Aβ treatment. Although no difference in OCR was observed in cells transfected with scramble shRNA and PU.1 shRNA and the control group, the OCR in the PU.1 shRNA-treated group was slightly lower than in the group treated with scramble shRNA alone (Figure 8B-C). To compare the effect of PU.1 versus NOX4 silencing in Aβ_1-40_-induced oxidative stress, ROS and mtROS levels in primary mouse RPE cells after siRNA treatment were determined. Both total ROS and mtROS levels were decreased in NOX4 siRNA group compared with the negative control. However, PU.1 depletion decreased the total ROS production to a greater extent than NOX4 silencing in Aβ_1-40_-treated primary RPE cells (Supplementary Figure S5A-D).

**Figure 9:**
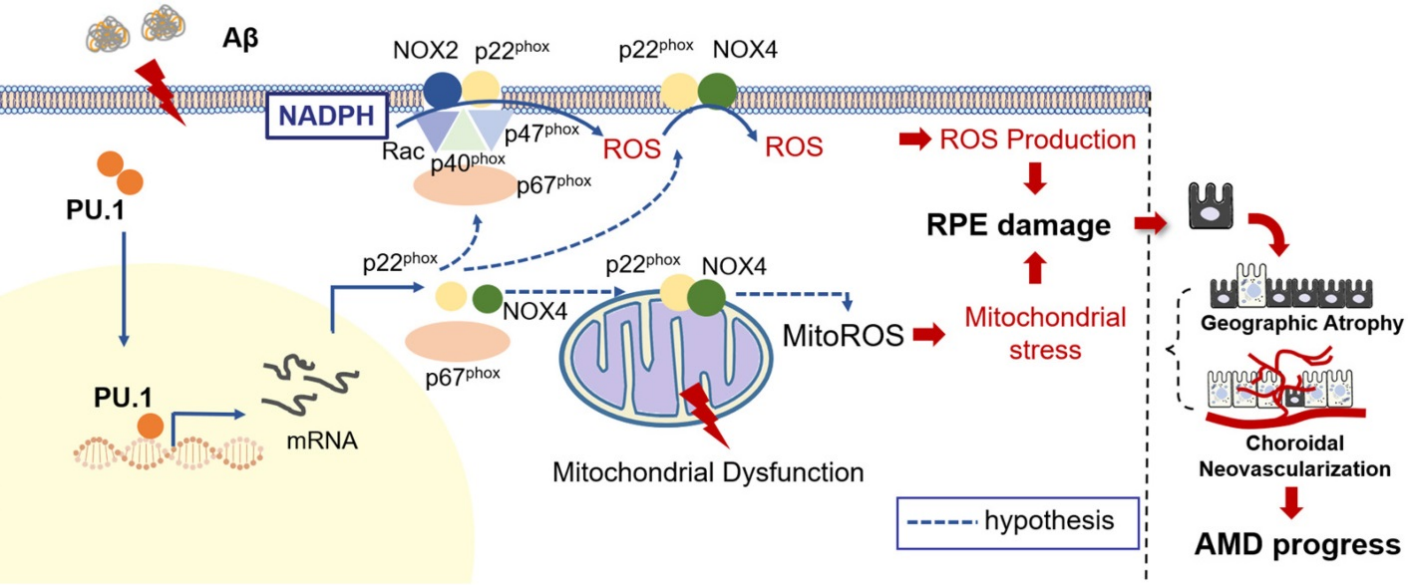
** Schematic representation of the Aβ_1-40_-mediated pathological process obtained by integrating the data obtained in this study.** PU.1 transcriptionally regulated the expression of NADPH oxidases, especially the NOX4-p22^phox^ complex, in Aβ_1-40_-mediated RPE injury in the progression of AMD.

Due to the reduced oxidative stress level and partially restored mitochondrial function, pathological changes, especially the multiple patchy deposits, were less notable in mice treated with Aβ_1-40_ and PU.1 shRNA (Figure 8E), and the ONL thickness was recovered (Figure 8H-I). Besides these pathological changes, the b-wave amplitude in the ERG of Aβ_1-40_- and PU.1 shRNA-treated groups was higher than in the Aβ_1-40_- and scramble shRNA-treated groups (Figure 8F). Though both the ONL thickness and b-wave amplitude were decreased in the PU.1 shRNA-treated group compared with the shRNA scramble-treated group, these retinal abnormalities, along with the decreased OCR, may be due to the negative effect of lentivirus subretinal injection or to inhibition of the broad biological functions of PU.1. Therefore, although PU.1 silencing caused slight damage to the retina, PU.1 downregulation still largely reversed the damaging effects of Aβ_1-40_, indicating that PU.1 is a crucial mediator of Aβ_1-40_-induced RPE degeneration through its regulation of oxidative stress. A schematic diagram of the role of PU.1 in the pathogenesis of Aβ_1-40_-mediated, NOX4-p22^phox^-related oxidative stress is illustrated in Figure 9.

## Discussion

Oxidative stress and mitochondrial dysfunction, indicated by elevated total ROS and mtROS levels, are considered crucial for age-related diseases, especially those characterized by Aβ-induced neuron degeneration, such as Alzheimer's disease and AMD. The OCT images of AMD patients at 10-year follow-up imply that the accumulation of drusen was associated with RPE injury and AMD progression. Our proteome analysis provided evidence that Aβ_1-40_-deposited RPE cells are characterized by NOX expression, especially the NOX4-p22^phox^ complex, which mediates ROS production and mitochondrial dysfunction regulated by the transcriptional regulator PU.1. Accordingly, inhibition of PU.1 expression by subretinal injection of PU.1 shRNA lentivirus attenuated oxidative stress levels and RPE dysfunction in Aβ-treated mice, indicating that PU.1 intervention could restore Aβ-mediated RPE damage by reducing ROS production and mitochondrial dysfunction.

RPE cells are nourished by the choriocapillaris and RPE degeneration might result in secondary choriocapillaris loss 37. Choriocapillaris loss and flow impairment in AMD presumably cause RPE hypoxia, which may lead to oxidative stress and the production of vascular endothelial growth factor. As shown in our study, there is a spatial relationship between drusen and choriocapillaris loss; this has been confirmed in a specific phenotype associated with the development of late AMD, namely, reticular pseudo-drusen (RPD), which has been shown to be more tightly associated with reduced choriocapillaris flow 38. RPE/Bruch's membrane/choriocapillaris complex degeneration is recognized to play an essential role in AMD development. However, the principal cause of this degeneration and its relationship between drusen deposition, RPE oxidative stress, and choriocapillaris loss still remain to be elucidated.

Recent studies have indicated the important role of oxidative stress-related processes in RPE degeneration along with the development of AMD 39-41. Several oxidative stress-induced RPE damage models, especially H_2_O_2_-treated RPE cells in which the pathological mechanism is intimately associated with the Nrf2 signaling pathway, have been established 40, 41. However, the role of H_2_O_2_ in AMD remains controversial, and H_2_O_2_-treated RPE cells are often used to verify the efficiency of newly discovered antioxidants. Besides, sodium iodate (NaIO_3_)-induced retinal injury is also reported recently to be an oxidant-related damage model, which is shown to be a murine model of müller cell-dominated retinal fibrosis rather than RPE degeneration 42. As per our clinical findings and previous research 9, the phenotypes of Aβ_1-40_-deposited RPE cells resembled those observed in AMD. In contrast to H_2_O_2_/NaIO_3_-induced oxidative stress, the one stimulated by Aβ_1-40_ was mediated by NADPH oxidases, especially the NOX4-p22^phox^ complex, as shown by our data. Other studies also focused on NADPH oxidases involved in Aβ-induced RPE injury 34. Generally, p22^phox^ forms a complex with NOX1, NOX2, NOX3, or NOX4 to stabilize their expression 43. Among them, the heterodimer formed with NOX2 (formerly called gp91^phox^) is the best-known NOX complex, the cytochrome b558 complex, located at the cell membrane and functions in Aβ-activated microglia in the central nervous system 44, 45. The ROS elevation by p22^phox^ is not attributed to a particular member of the NADPH oxidase family. Among this family, the heterodimer composed of p22^phox^ and NOX4 is a unique complex that produces H_2_O_2_ 46. NOX4-derived H_2_O_2_ has been implicated in retinal neovascularization 47, and NOX4-mediated oxidative stress seems to promote the progression of CNV (late-stage AMD) 48. What's more, NOX4 was also reported to regulate cyclic stretch induced RPE cells injury in vitreomacular adhesion related AMD 49. Notably, CM-H2DCFDA staining, the method we used to detect the total ROS level, is more sensitive to oxidation by H_2_O_2_ than by superoxide (O_2_^•-^), which is primarily produced by NOX2 50. Thus, the detection of a CM-H2DCFDA signal in our study may support a different mechanism of Aβ_1-40_-induced RPE oxidative stress due to H_2_O_2_ produced by the p22^phox^-NOX4 heterodimer, suggesting that Aβ_1-40_-induced RPE degeneration is a more reasonable model of AMD-like RPE damage than RPE injured by H_2_O_2_ incubation.

Besides NOX, mitochondria are another major source of ROS production. Research on RPE cells in AMD donor eyes indicated a bioenergetic crisis that may contribute to AMD pathology 51. Interestingly, recent investigations have shown that NOX4 expression is related to mtROS production, resulting in mitochondrial oxidative stress 20, 52. In the current study, we found that both mtROS and NOX4 were augmented in Aβ_1-40_-stimulated RPE cells, suggesting a link between mtROS production and NOX4. However, among different locations of NOX4, only mitochondrial NOX4 was reported to produce mtROS 53, whereas NOX4 predominantly localized in the ER generated ER stress-induced ROS 54. NOX4 has also been identified in other locations in various cell lines, such as the nucleus, cytoskeleton, and plasma membrane of HEK293 cells or vascular cells 55. The subcellular distribution of NOX4 may be cell type-specific, and its location and association with mtROS in Aβ_1-40_-deposited RPE cells require further investigation. In this context, another important discovery in this study is that Aβ_1-40_-induced RPE ROS production was derived from multiple sources. Although cytotoxic effects of Aβ_1-40_ on RPE cells have been reported earlier 56, our data showed that ROS elevation and mitochondrial damage may not be the features of Aβ-mediated RPE apoptosis following Aβ_1-40_ treatment at the concentration of 2 μM.

NOX4 and mtROS also exhibit a clear link with inflammation. Mitochondrial NOX4 was reported to promote NLRP3 inflammasome activation through the fatty acid oxidation (FAO) pathway 57, and mtROS was shown to regulate Aβ-induced proinflammatory cytokine expression 35. Inflammatory cytokine production affected TLR5-NOX4-ROS signaling-mediated NF-κB activation 18, which was demonstrated to be a critical regulator in RPE-mediated inflammation, as reported in our previous study 7. Additionally, TLRs are known to function as mediators of Aβ signaling in microglia 58. These findings indicated the potential of NOX4 inhibitors, such as GKT137831, which is currently in clinical development as an efficient pharmacological strategy 59. However, inhibition of NOX4 expression alone may not be sufficient; thus, we investigated additional upstream regulatory targets.

PU.1, an ETS family transcription factor, has been shown to regulate the expression of NOX2 and p22^phox^, p47^phox^ and p67^phox^ subunits in immune cells, such as lymphocytes, dendritic cells, and myelomonocytic cells 60, 61. However, not much is known about the role of PU.1 in RPE cells, its relationship with NOX4, and its association with NADPH oxidases. Our data demonstrated that the transcription factor PU.1 was critical for NOX4, p22^phox^, and p67^phox^ but not NOX2 or p47^phox^ in Aβ_1-40_-deposited RPE cells. These contradictory results were possibly due to the differences in cell types and the source of stimuli. So far, most PU.1-related retinal studies have focused on fundus inflammation, such as uveitis and photoreceptor dystrophy, and not on oxidative stress, mainly because PU.1 is a master regulator of myeloid and lymphoid lineage generation and the immune system 62, 63. We have previously reported that Aβ-induced damage was closely related to proinflammatory cytokine production, and ChIP-seq analysis showed that PU.1 regulated the expression of genes relevant to Aβ-activated microglia 64. Therefore, further investigation is required to elucidate whether PU.1 is related to RPE cell inflammation and whether epigenetic modification via histone deacetylase 1 occurs in addition to transcriptional regulation, as reported in a previous study 65.

Our study demonstrated a decrease in NOX-mediated ROS levels and the partial recovery of Aβ_1-40_-induced damage to retinal function induced by PU.1 silencing, suggesting that PU.1 is an upstream target of Aβ_1-40_-induced retinal degeneration. Nevertheless, it should be noted that PU.1 knockdown resulted in slight injury to retinal function for unknown reasons, which may be related to inhibition of the normal function of PU.1 or its intersection with other protective pathways. Thus, we targeted its downstream protein NOX4. However, the results revealed that PU.1 depletion could decrease both total ROS and mtROS levels to a greater extent than NOX4 silencing in Aβ_1-40_ treated RPE cells. It was probably because not only NOX4 but also p22^phox^ and p67^phox^ expression were PU.1-dependent in Aβ_1-40_-treated RPE cells and low expression level of p22^phox^ further led to the loss of NOX4 and NOX2 activity 66, 67. Despite the strong effect of PU.1 depletion on the functional integrity of RPE cells, given the multi-faceted effects of Aβ_1-40_ on RPE oxidative stress indicated by proteomic profiling, it would be worthwhile to identify possible upstream regulators of PU.1 as potential therapeutic target(s).

## Conclusions

Our proteomic analysis findings reveal that NADPH oxidases-mediated oxidative stress, including mitochondrial oxidative stress and dysfunction, is an essential biological process in Aβ_1-40_-mediated RPE degeneration. The expression of NADPH oxidases, especially the NOX4-p22^phox^ complex, is transcriptionally regulated by PU.1. The inhibition of PU.1 may restore partial retinal function by decreasing oxidative stress and relieving mitochondrial dysfunction, suggesting its potential as a novel target for AMD treatment.

## Supplementary Material

Supplementary figures and tables.

Supplementary table S1.

## Figures and Tables

**Table 1:** Follow-up examination of early AMD patients with or without drusen

Drusen presence	AMD stage at final visit				*P*-value*
	Early/Intermediate AMD (stable)		Advanced AMD (progress)		
	No. of eyes	%	No. of eyes	%	
no Drusen	12	47.8	1	4.2	0.000237
with Drusen	11	52.2	23	95.8	

*Fisher's exact test; AMD: age-related macular degeneration.

## Abbreviations

Aβ: Amyloid β
AREDS: Age-Related Eye Disease Study
AMD: age-related macular degeneration
ChIP: chromatin immunoprecipitation
CNV: choroidal neovascularization
Co-IP: co-immunoprecipitation
DEP: differentially expressed protein
ER: endoplasmic reticulum
ERG: electroretinogram
ETS: E26 transformation-specific
FAO: fatty acid oxidation
FAZ: foveal avascular zone
GA: geographic atrophy
GO: gene ontology
GSEA: gene set enrichment analysis
H&E: hematoxylin & eosin
INL: inner nuclear layer
IVL: intravitreal
MS: mass spectrometry
mtROS: mitochondrial reactive oxygen species
NOX: NADPH oxidase
OCR: oxygen consumption rate
OCT: optical coherence tomography
OCTA: optical coherence tomography angiography
PBS: phosphate-buffered saline
PRM: parallel reaction monitoring
PPI: protein-protein interaction
qRT-PCR: quantitative real-time PCR
ROS: reactive oxygen species
RPD: reticular pseudo-drusen
RPE: retinal pigment epithelium
SD-OCT: spectral domain OCT
shRNA: small hairpin RNA
SSADA: split-spectrum amplitude-decorrelation angiography
ONL: outer nuclear layer
TEM: transmission electron microscopy
TLR: Toll-like receptor
TMT: tandem mass-tagged
TOM: topological overlap matrix
WGCNA: weighted gene co-expression network analysis
