# Supplementary material for: ROS production and mitochondrial dysfunction driven by PU.1-regulated NOX4-p22phox activation in Aβ-induced retinal pigment epithelial cell injury
Source: Theranostics. 2020 Sep 19;10(25):11637–55. doi: 10.7150/thno.48064 (PMC7546003; doi:10.7150/thno.48064)
Supplement: Supplementary file 1 — Supplementary figures and tables. [file thnov10p11637s1.pdf]

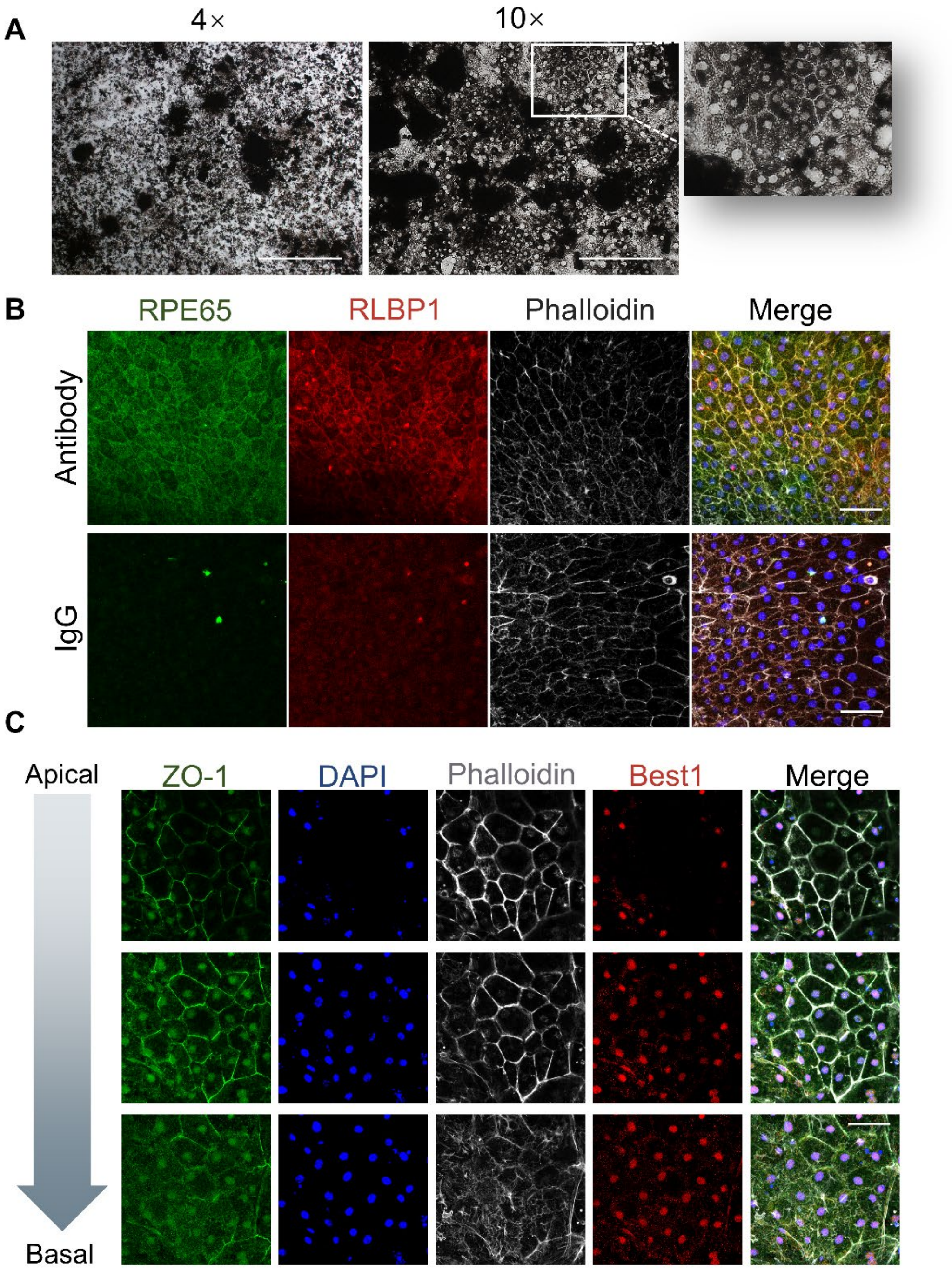

Supplementary Figure S1: Identification of Primary mouse RPE cells.

(A) The pigmented cells were investigated by inverted light microscopy at 80% confluency (4×) and showed a typical hexagonal morphology (10×). Scale bars = 500 µm for the images labeled "4×", scale bars = 200 µm for the images labeled "10×".

(B) Confocal images of immuno-stained primary RPE cells using specific markers phalloidin (white), RPE65 (green) and RLBP1 (red). IgG isotype antibodies were used as negative control. Scale bars = 500 μm.

(C) ZO-1, Phalloidin and Best 1-immuno-stained primary RPE cells at different layers. Scale bars = 50 μm.

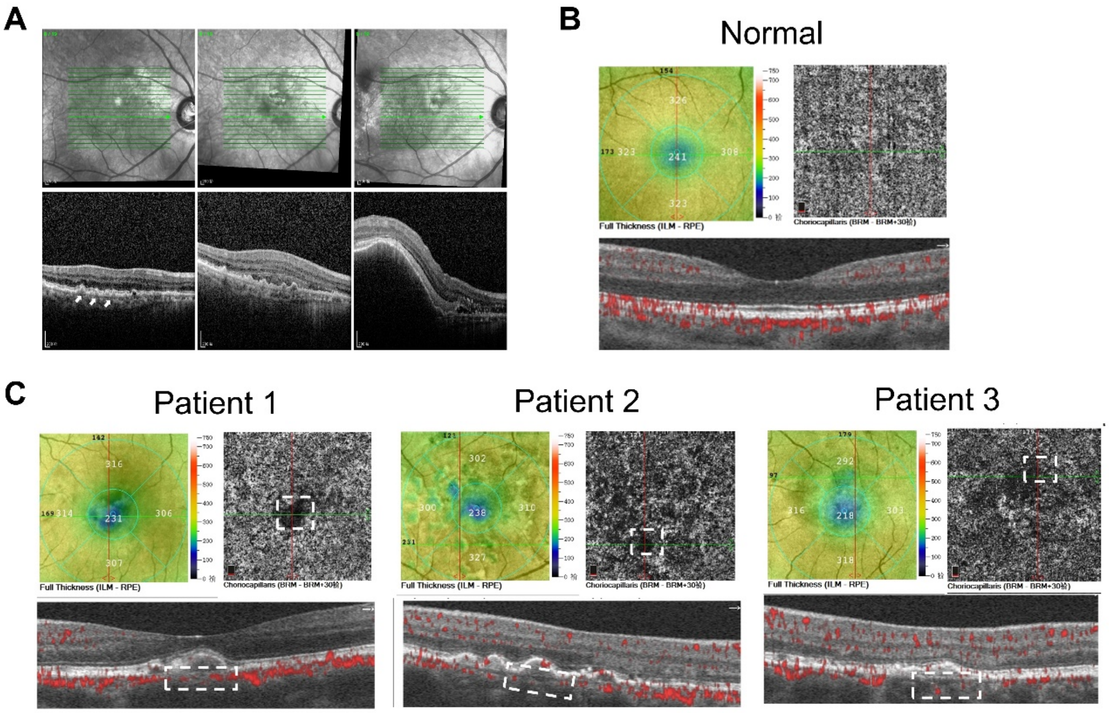

Supplementary Figure S2: Representative images showing the progression of drusen deposition to an advanced stage of AMD in patients.

(A) Baseline and follow-up OCT images from the right eye of an 82-year-old man initially diagnosed with early dry AMD in 2012. Images from a cross-sectional B-scan (left panel) conducted in June 2012 showed multiple drusen deposits (white arrows). In December 2012, follow-up OCT (middle panel) images revealed increased volumes of multiple drusen compared with their volumes in former scans, accompanied by subretinal hyperreflexia and exudation, while CNV lesion/advanced AMD was observed at the same position in OCT scans taken when he returned in January 2015 (right panel).

(B, C) OCT angiograms of the choriocapillaris (3 × 3 mm) and the full thickness (inner limiting membrane-retinal pigment epithelium) of 1 normal eye and 3 early AMD eyes with the presence of drusen. Areas of decreased choriocapillaris signaling are marked by white squares (top). Cross-sections of OCT B-scans at green lines and the corresponding areas of decreased choriocapillaris signaling under drusen are displayed in C (bottom, white square).

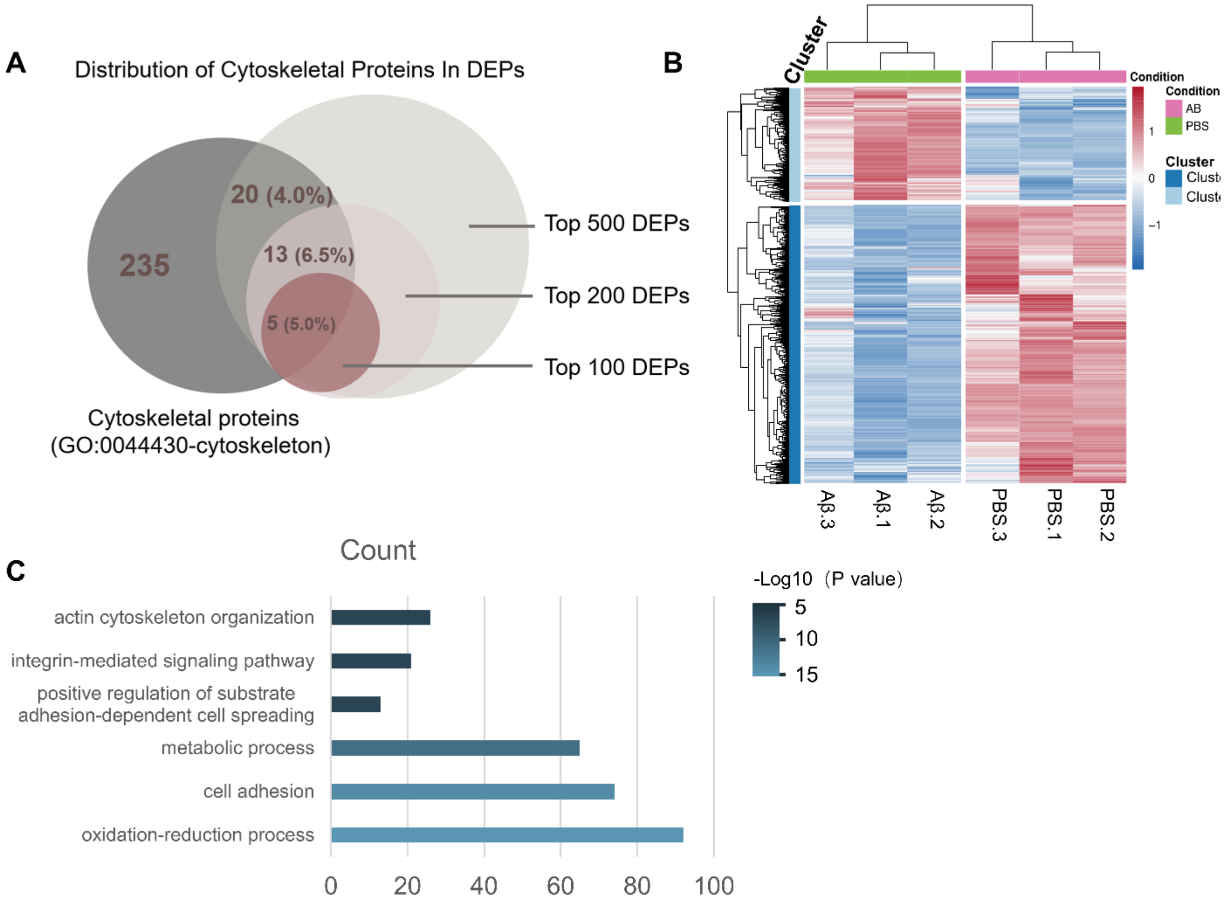

Supplementary Figure S3: Changes of protein expression pattern after A$\beta_{1\text{-}40}$ treatment.

(A) Distribution of cytoskeletal proteins (according to GO: 0044430-cytoskeleton) in top up-regulated differentially expressed proteins (DEPs) ($\log_2$ (fold change [FC]) > 0.5 or < -0.5 and P ≤ 0.05) ranked by absolute $\log_2$FC.

(B) GO enrichment analysis of total DEPs (absolute $\log_2$FC > 0.5 and P ≤ 0.05) and major GO terms are enriched in cytoskeleton-related terms.

(C) Heatmap of the gene expression pattern in the MEblue module: Genes in the MEblue module were grouped into “cluster 1” (985 proteins) and “cluster 2” (411 proteins) based on the expression.

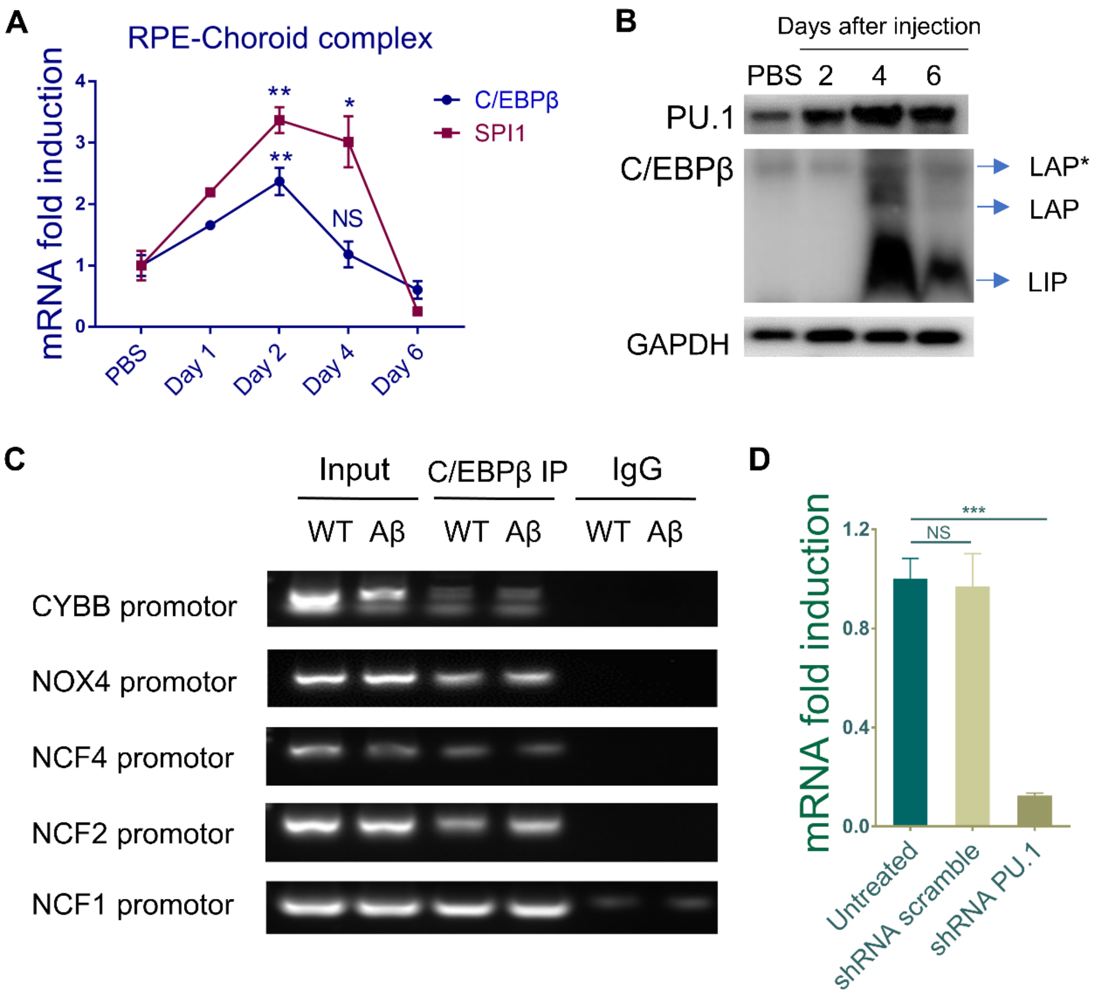

Supplementary Figure S4: Increased expression of PU.1/Spi1 and C/EBPβ at both the mRNA and protein levels.

(A) mRNA levels of Spi1 and C/EBPβ at different times after RPE-choroid complex exposure to $A\beta_{1-40}$ were evaluated by qRT-PCR. The mRNA levels were standardized to those of a housekeeping gene.

(B) PU.1 and C/EBPβ expression in mouse RPE cells in response to 2, 4, and 6 days of $A\beta_{1-40}$ treatment. LAP, liver-enriched activator protein; LIP, liver-enriched inhibitory protein; both LAP and LIP are isoforms of C/EBPβ. Blots for PU.1 and GAPDH were identical to those shown in Figure 8A.

(C) ChIP analysis of the association of C/EBPβ with the CYBB, NOX4, NCF4, NCF2, and NCF1 gene promoters was performed 12 h after Aβ stimulation. The immunoprecipitated DNA was then analyzed by semiquantitative

PCR.

(D) Measurement of lentiviral PU.1 shRNA interference efficiency in primary mouse RPE cells by qRT-PCR.

All data are presented as the mean ± SEM; NS = nonsignificant, *$P < 0.05$, **$P < 0.01$, Student's t-test.

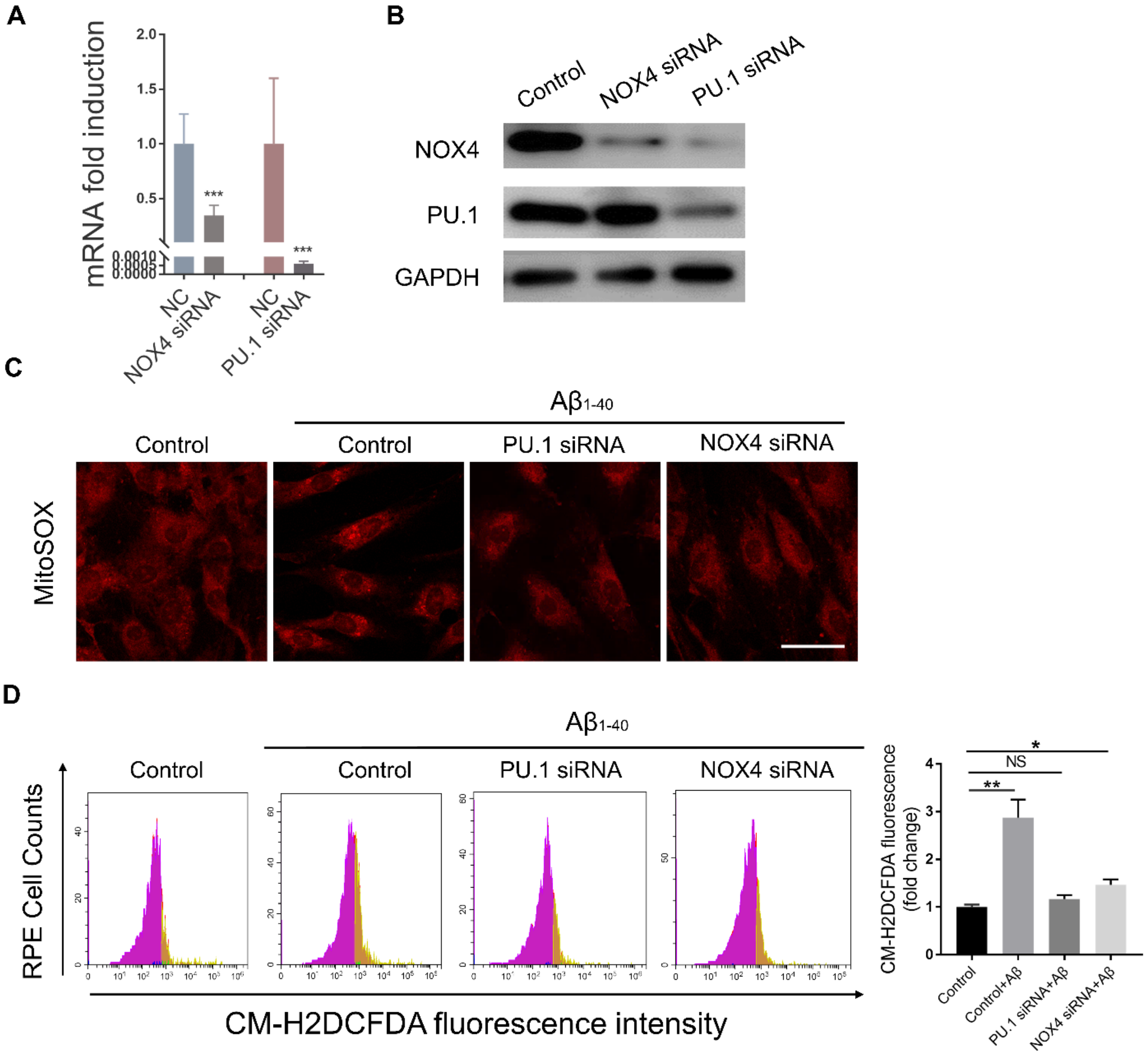

Supplementary Figure S5: PU.1 and NOX4 targeted knockdown decreased ROS levels in primary RPE cells.

(A, B) Measurement of PU.1 and NOX4 siRNA interference efficiency in primary mouse RPE cells by qRT-PCR (A), Western blot (B). Primary RPE cells transfected with non-targeting siRNA were used as the negative control (NC).

(C) Representative images of MitoSOX Red-stained primary mouse RPE cells transduced with Control/PU.1/NOX4 siRNA at 12 h after $A\beta_{1\text{-}40}$ treatment. Scale bar = 50 μm.

(D) The CM-H2DCFDA fluorescence intensity was detected in primary mouse RPE cells transduced with

Control/PU.1/NOX4 siRNA followed by $A\beta_{1-40}$ (2 μM) incubation for 12 h.

All data are presented as the mean ± SEM; ***P < 0.001, Student's t-test were performed using GraphPad Prism software.

Table S2: Primers for PCR and ChIP

Primers pairs for qRT-PCR		
CYBA	5’-TGCCAGTGTGATCTATCTGCT-3’	5’-TCGGCTTCTTTCGGACCTCT-3’
NCF1	5’-ACACCTTCATTCGCCATATTGC-3’	5’-CCTGCCACTTAACCAGGAACA-3’
NCF2	5’-GGAGAAGTACGACCTTGCTATCA-3’	5’-ACAGGCAAACAGCTTGAACTG-3’
NCF4	5’-GTCATCGAGGTCAAAACAAAAGG-3’	5’-TCCGAGTCTCAGCGATCTCTT-3’
CYBB	5’-AGTGCGTGTTGCTCGACAA-3’	5’-GCGGTGTGCAGTGCTATCAT-3’
NOX4	5’-CCCAAGTTCCAAGCTCATTTCC-3’	5’-TGGTGACAGGTTTGTTGCTCCT-3’
Spi1	5’-TTACAGGCGTGCAAAATGGAA-3’	5’-GACGTTGGTATAGCTCTGAATCG-3’
ChIP primers for PU.1		
NCF2	5’-CCAACTACACGAATGTGCTAGATGA-3’	5’-ATAAGGCCATTGTGTTTTGATCAGA-3’
NCF4	5’-TTAGGAATGAATGGCAGGTC-3’	5’-CCACCCACCATAGAGCAG-3’
NOX4	5’-TGTCTCCTGTAACATTCCCTG-3’	5’-TAAAGAAAGGAGACTATCCAAAGCC-3’
CYBA	5’-TCTTCCCCAGGTTCCTAAA-3’	5’-ACCTGAGTGGCAATGATGTA-3’
